# 50/50 Expressional Odds of Retention Signifies the Distinction between Retained Introns and Constitutively Spliced Introns in *Arabidopsis thaliana*

**DOI:** 10.3389/fpls.2017.01728

**Published:** 2017-10-09

**Authors:** Rui Mao, Chun Liang, Yang Zhang, Xingan Hao, Jinyan Li

**Affiliations:** ^1^College of Information Engineering, Northwest A&F University, Yangling, China; ^2^Department of Biology, Miami University, Oxford, OH, United States; ^3^Department of Computer Sciences and Software Engineering, Miami University, Oxford, OH, United States; ^4^State Key Laboratory of Crop Stress Biology for Arid Areas, College of Plant Protection, Northwest A&F University, Yangling, China; ^5^Advanced Analytics Institute, University of Technology Sydney, Sydney, NSW, Australia

**Keywords:** retained introns (RIs), constitutively spliced introns (CSIs), high-throughput next-generation RNA sequencing (RNA-Seq), distinguishable features, random forest, intronic splicing silencers (ISSs), intronic splicing enhancers (ISEs)

## Abstract

Intron retention, one of the most prevalent alternative splicing events in plants, can lead to introns retained in mature mRNAs. However, in comparison with constitutively spliced introns (CSIs), the relevantly distinguishable features for retained introns (RIs) are still poorly understood. This work proposes a computational pipeline to discover novel RIs from multiple next-generation RNA sequencing (RNA-Seq) datasets of *Arabidopsis thaliana*. Using this pipeline, we detected 3,472 novel RIs from 18 RNA-Seq datasets and re-confirmed 1,384 RIs which are currently annotated in the TAIR10 database. We also use the expression of intron-containing isoforms as a new feature in addition to the conventional features. Based on these features, RIs are highly distinguishable from CSIs by machine learning methods, especially when the expressional odds of retention (i.e., the expression ratio of the RI-containing isoforms relative to the isoforms without RIs for the same gene) reaches to or larger than 50/50. In this case, the RIs and CSIs can be clearly separated by the Random Forest with an outstanding performance of 0.95 on AUC (the area under a receiver operating characteristics curve). The closely related characteristics to the RIs include the low strength of splice sites, high similarity with the flanking exon sequences, low occurrence percentage of YTRAY near the acceptor site, existence of putative intronic splicing silencers (ISSs, i.e., AG/GA-rich motifs) and intronic splicing enhancers (ISEs, i.e., TTTT-containing motifs), and enrichment of Serine/Arginine-Rich (SR) proteins and heterogeneous nuclear ribonucleoparticle proteins (hnRNPs).

## Introduction

Alternative splicing is a biological mechanism that gives rise to different transcript isoforms from the same genes. The expression levels of alternatively spliced isoforms of a gene can change at different growth stages or under different environmental conditions. The prevalence of alternative splicing in plants has been investigated recently using high-throughput next-generation RNA sequencing (RNA-Seq) technologies (Marquez et al., 2012; Thatcher et al., 2014; Conesa et al., 2016). Compared with previous studies (Severing et al., 2011; Syed et al., 2012), these studies revealed higher proportions of genes that show alternative splicing in different species. For example, around 40% of the multi-exon genes of *Zea mays* (Thatcher et al., 2014), 63% of the multi-exon genes in *Glycine max* (Shen Y. et al., 2014), and 60% of the multi-exon genes in *Arabidopsis thaliana* (Marquez et al., 2012) were found to have alternative splicing events. This trend of increasing prevalence is mainly attributed to the richer data obtained by RNA-Seq. In another study through RNA-Seq profiling, Klepikova et al. identified 37,873 novel splice junctions in Arabidopsis which are not included in TAIR10 database (Klepikova et al., 2016). RNA-Seq has now opened up great opportunities to understand many unknown transcriptome landscapes in plants.

Intron retention is one of the most prevalent events of alternative splicing in plants. If an intron is always spliced out from all the isoforms of the gene, it is known as a constitutively spliced intron (CSI). However, an intron of a gene that can be retained in one or more isoforms whereas spliced out in other isoforms is called a retained intron (RI). The prevalence of intron retention has been seen in many plants, including *Oryza sativa* (Campbell et al., 2006), *A. thaliana* (Filichkin et al., 2010), *Gossypium raimondii* (Li Q. et al., 2014), *Sorghum bicolor* (Panahi et al., 2014), *Zea mays* (Thatcher et al., 2014), *Brachypodium distachyon* (Vitulo et al., 2014), and *Medicago truncatula* (Zhang et al., 2016), with a 45.1, 64.1, 40, 41, 58, 55.5, and 51.3% prevalence over the alternative splicing events, respectively. Accumulating evidences have also suggested that intron retention can regulate specific abscisic acid (ABA) signaling, affect other developmental processes, and strengthen the response to environmental stresses and conditional signals (Reddy et al., 2013; Panahi et al., 2015; Wang et al., 2015). To understand a wider range of characteristics and features of the RIs in Arabidopsis, we developed a new computational pipeline for discovering new RIs from multiple RNA-Seq datasets acquired under different developmental stages or environmental conditions.

**Figure 1:**
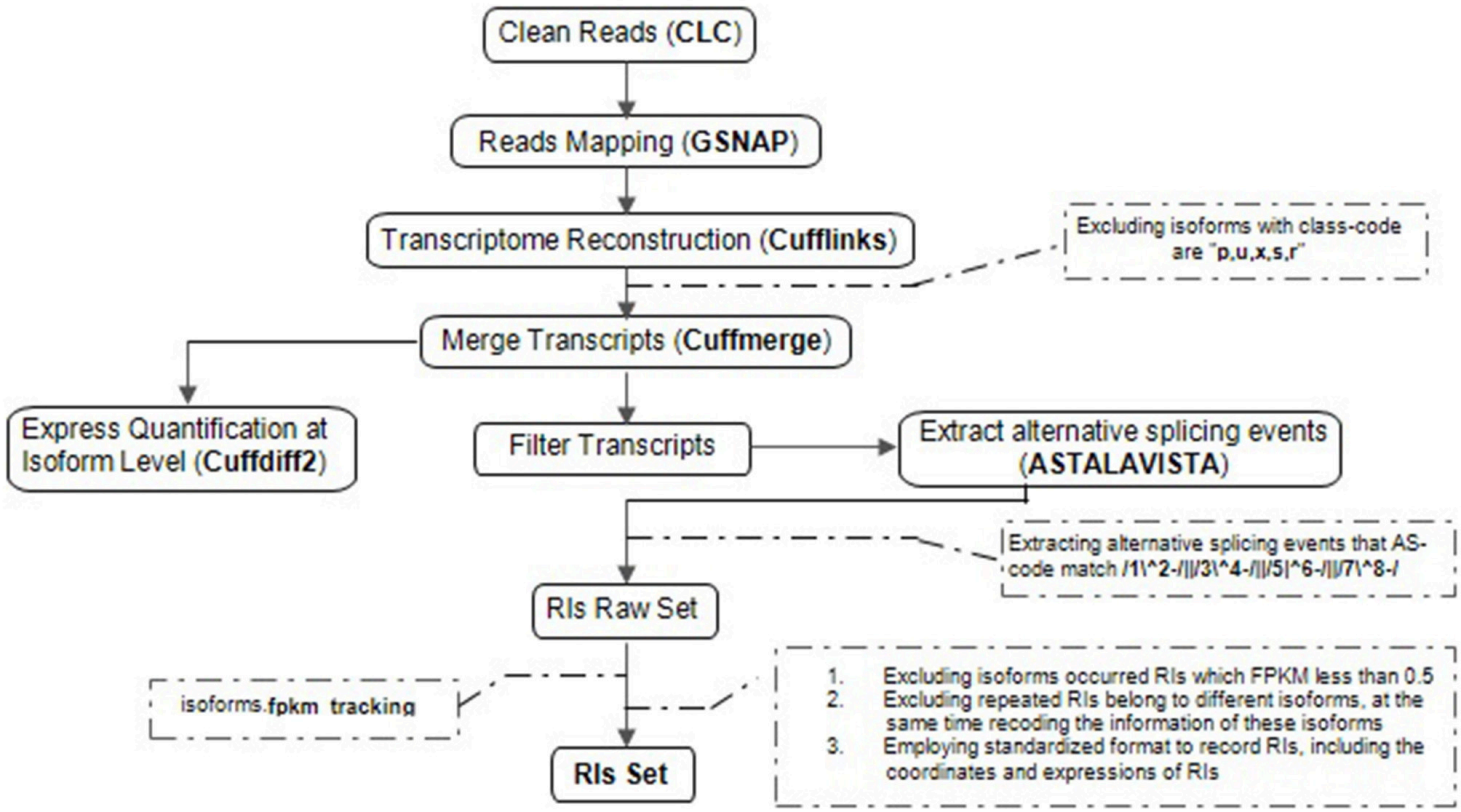
The computational pipeline for the identification of RIs. Overview of the steps involved in clean reads, reads mapping, transcriptional reconstruction, RIs extraction and express quantification at isoform level, specific procedural methods, and corresponding software's are described in order to identifying reliable RIs for each class of RNA-Seq dataset.

Our computational pipeline consists of five main steps for the accurate identification of RIs and CSIs from multiple RNA-Seq datasets (Figure 1). At first, adaptor sequences and low-quality reads within the raw RNA-Seq reads are trimmed or removed using Trim sequences tool in CLC Genomics Workbench. Secondly, GSNAP is used to map clean reads to the reference genome (Wu and Nacu, 2010). Unlike Tophat (De Bona et al., 2008), GSNAP adopts a “seed and extend” algorithm, and can detect known and novel splice junctions in individual reads more accurately. Thirdly, based on the high-quality genome sequences of Arabidopsis, Cufflinks, a popular genome-guided transcriptome analysis tool, is utilized to reconstruct a transcriptome (Trapnell et al., 2010). Although Cufflinks reports the minimal number of compatible isoforms with lower sensitivity, it can obtain a higher accuracy of transcripts in comparison with other similar tools like Scripture (Guttman et al., 2010). Fourthly, cuffdiff2 is used to estimate expression quantification at the isoform level because of the best compatibility (Trapnell et al., 2013). It not only directly utilizes the merging transcriptomes from differential conditions with cuffmerge but also effectively controls the over-dispersion problems of biological replicates. Lastly, we developed a software package (RIs_CSIs_ID) to merge separate results and conduct other downstream computational tasks required for comprehensive and accurate identification of RIs and CSIs.

We are also interested in the classification and distinction between the RIs and CSIs by machine learning methods, and proposed a new feature vector to describe both two types of introns for more accurate classification. Conventional features include linear sequence features of intron, short frequent sequence motifs, and other splice sites-related features (Mao et al., 2014). In particular, we introduced the use the expression of intron-containing isoforms as a new feature to describe introns in addition to the conventional features. Under the representation by the new feature vector, RIs and CSIs can be distinctively separated by machine learning methods. To see the best classification between RIs and CSIs, we focused on some subsets of RIs that have high expressional odds of retention. The expressional odds of retention for a retained intron in a gene are defined as the expression ratio of the RI-containing isoforms relative to the isoforms without the RI for the same gene. When the expressional odds of retention reaches to or larger than 50/50, the RIs and CSIs can be clearly separated by the Random Forest learning algorithm (Nair et al., 2013) with an outstanding performance of AUC 0.95 on average. By incorporation expression data, our classification performance had a significant improvement in comparison with those previously reported methods (Eichner et al., 2011; Mao et al., 2014).

It is well-known that splice sites, branch point and cis-regulatory elements have impacts on pre-mRNA splicing (Zhang et al., 2011; Wittkopp and Kalay, 2012; Meyer et al., 2015). However, the relevant features and conservative motifs are waiting to be resolved. For example, the cis-regulatory elements located in the introns are usually considered as intronic splicing enhancers or silences (ISEs or ISSs), but what frequent motifs are species-specific ISEs and ISSs remains unknown. Based on the newly found RIs and CSIs, we detected the signal strength and the similarity between the flanking sequences of splice sites, the conservative branch point sequence motifs, ISSs, ISEs, and the enrichment of SR (Serine/Arginine-Rich) proteins and hnRNPs (heterogeneous nuclear ribonucleoparticle proteins). We discovered those contrasting or determinative features have outstanding differences between RIs and CSIs, indicating their involvement in alternative splicing processes.

## Materials and methods

### RNA-seq datasets

We collected 18 RNA-Seq datasets of Arabidopsis from SRA (http://www.ncbi.nlm.nih.gov/sra/, the accession numbers of these SRA data are shown in Table 1). These datasets were represented as 6 different groups, which were sampled in different developmental stages or under different environmental conditions. The first group has two datasets, SRR360152 and SRR360154 (SRP009136), containing RNA-Seq profiles of Arabidopsis tissues under the conditions 10-d seedlings and flowers with a 1:1 ratio (Marquez et al., 2012). The second group also has two datasets, SRR1104149 and SRR1104886 (SRP035230), containing RNA-Seq profiles of Arabidopsis tissues at the stage of inflorescent meristem (Wang et al., 2014). The other 14 datasets contain RNA-Seq profiles of Arabidopsis at environmental stress responses to cold, heat, salt and drought (Filichkin et al., 2010), which are organized into four groups accordingly. So, the 18 datasets are organized into six groups and denoted as Sample1, Sample2, Sample3, Sample4, Sample5 and Sample6, which, respectively, match with the conditions “10-d seedlings and flowers with a 1:1 ratio,” “inflorescent meristem,” “cold,” “heat,” “salt” and “drought.” Other details of these RNA-Seq datasets are listed in Table 1.

**Table 1:** Data sources of RNA-Seq.

**Classes**	**ID in SRA**	**Tissues or growth conditions**	**Sequencing instrument**	**The length of reads (bp)**	**Sequencing types**	**The Percentage of aligned mapping (Q ≥ 20) (%)**
Sample1	SRR360152	10-d seedlings and flowers mixed in a 1:1 ratio	Illumina Genome Analyzer II	76	Paired	98.79
	SRR360154					98.78
Sample2	SRR1104149	Inflorescent meristem	AB SOLiD System 3.0	50	Single	99.95
	SRR1104886					99.94
Sample3	SRR018179	Cold	Illumina Genome Analyzer II	36	Single	97.03
	SRR018180					99.77
	SRR018181					99.65
	SRR545949					91.12
Sample4	SRR018185	Heat	Illumina Genome Analyzer II	36	Single	92.83
	SRR018186					95.78
	SRR018187					99.86
	SRR545950					91.40
Sample5	SRR019206	Salt	Illumina Genome Analyzer II	36	Single	88.27
	SRR019207					87.73
	SRR545952					85.61
Sample6	SRR019209	Drought	Illumina Genome Analyzer II	36	Single	92.58
	SRR019210					92.86
	SRR545953					90.81

*All samples of RNA-Seq in analysis are divided into six classes (denoted as Sample1, Sample2, Sample3, Sample4, Sample5 and Sample6) based on different issues and conditions. The percentage of aligned mapping illustrates the percents of the aligned mapping reads with the phred quality score (Q) more than 20 (Q ≥ 20) after running gsnap*.

### Extraction of RIs and CSIs from multiple RNA-seq datasets

Extraction of RIs from multiple RNA-Seq datasets is a complicated process, including five steps described in Figure 1.

#### Clean reads

To remove low-quality and adaptor sequences within the raw reads, Trim sequences tool in CLC Genomics Workbench was employed for cleaning raw reads. (i) Trimming adapter fragments off raw sequence reads. (ii) Trimming reads with N bases over 10% (N is ambiguous base or nucleotide). (iii) Trimming reads with the Phred quality scores (Q) < 13. In Equation (1), *e* is the base-calling error probability, i.e., *Q* value of 13 is equivalent to 0.05 *e* value. These trimming steps ensure not only all clean reads without low-quality bases left for downstream analyses but also the error recognition rate of nucleotides in clean reads no more than 0.05.

(1) $$\begin{array}{r} \text{Q}=-10\log_{10}\left(e\right) \end{array}$$

#### Reads mapping

GSNAP v2016-04-04 (http://research-pub.gene.com/gmap/) can detect known and novel splice junctions in individual read, depending on the indexes of known splice junctions and the whole-gene sequence of Arabidopsis. So, we built these indexes in local server first, then set the parameter -N as 1 in order to find novel splice junctions. After reads mapping, the aligned reads that meet quality standards (Q ≥ 20) were counted. Aligned read percentages of all samples are illustrated respectively, in Table 1. The mean percentage of mapped reads is 94.60%, these mapped reads were used for the next transcriptome reconstruction.

#### Transcriptome reconstruction

For each SAM file after reads mapping, we independently assembled isoforms using Cufflinks v2.2.1 (http://cole-trapnell-lab.github.io/cufflinks/releases/v2.2.1/). It is known that plant introns are averagely shorter than their animal counterparts. We previously reported 96% introns were found within the range from 44 to 631 bp for TAIR10 (Mao et al., 2014). So we adjusted the parameter -I (-max-intron-length) from default 30,000 to 5,000 for Cufflinks. Meanwhile, the parameter -u (-multi-read-correct) was utilized to weigh reads mapping to multiple locations in the genome, and only highest ranking alignments were reported. Cuffmerge was employed to remove the redundant isoforms in different samples. Then, some potentially problematic isoforms with the class-code “p, u, x, s, or r” in contradiction with reference transcripts were filtered. Finally, we merged each two samples considering the complexity and space of computation, and obtained the possible sets of isoforms and genes, respectively, including 43,249 isoforms and 22,763 genes, 34,796 isoforms and 22,243 genes, 34,509 isoforms and 22,283 genes for Sample12, Sample34 and Sample56. In the following steps, we did systematic comparison among these isoforms according their coordinates in genome for all the six samples.

#### Express quantification at isoform level

Genes with multiple isoforms due to alternative splicing might have exons shared by different isoforms, which can lead to ambiguity in mapping fragments to isoforms and in quantifying expression of isoforms. Cuffdiff2 is efficient to reduce read assignment uncertainty and control the degree of overdispersion across biological replicates assisted by a beta negative binomial distribution. Reads per kilobase per million mapped reads (RPKM) is often used to normalize the isoform expression (Mortazavi et al., 2008).

(2) $$\begin{array}{r} \text{RPKM}=\frac{10^{9}C}{\text{NL}} \end{array}$$

In Equation (2), *C* represents the numbers of aligned reads in an isoform, N means the numbers of total aligned reads in a sample, L is the length of isoform. FPKM is very similar to RPKM, where fragments as a substitute instead of reads are counted out. RPKM is made for single-end RNA-Seq while FPKM is made for paired-end RNA-Seq. Here we unified with FPKM to quantify the isoform expression. A single fragment can correspond to one read for single-end RNA-Seq while to two reads for paired-end RNA-Seq. The files named isoforms.fpkm-tracking have recorded the expressions of our assembled isoforms.

#### Identification of RIs and CSIs

We used the ASTALAVISTA algorithm (Foissac and Sammeth, 2007) to identify all alternative splicing events from the GTF output files by cuffmerge (referring to File S1, Alternative_splicing_data). In this study, only intron retention events were examined. RIs can be directly identified by the record code of alternative splicing event. But these RIs include lots of redundant records because of the inner algorithm of ASTALAVISTA that is based on pairwise rather than global comparison. Moreover, no expression information of isoforms is shown on their records.

We proposed and implemented a software package (RIs_CSIs_ID) for the recognition of RIs from the results of ASTALAVISTA based on the comparison among all isoforms for the same genes. RIs_CSIs_ID is efficient to remove the redundant RI records of ASTALAVISTA. At the same time, it can add the expression information of isoforms and the coordinates of RIs to the final records, which is particularly critical when we compare differentiations of RIs among different tissues and growth conditions. Only does the RI-containing isoforms and the corresponding isoforms without the RI for the same gene are both expressed (FPKM no < 0.5), these RIs really happen in the biology and will be counted.

CSIs are always spliced out in all isoforms for one gene. If one intron is found in at least one mature isoform, it no longer is CSI. So the number of CSIs will be reduced with the increasing of novel RIs. RIs_CSIs_ID was also implemented for the recognition of CSIs. The user manual and open-source of RIs_CSIs_ID are freely available (please site https://pan.baidu.com/s/1o8DRbz8, also see File S2).

### Expressional odds of retention for the distinctions of various subsets of RIs from the set of CSIs

We divide the isoforms of a gene into two types when this gene contains introns which can be retained in some isoforms. One is the type of isoforms which contain the retained introns (RIs), and the other is the type of isoforms that do not have any of these introns. The “*expressional odds of retention*” of an intron is defined as the ratio of the FPKM of isoforms containing the retained intron, divided by the FPKM of the other isoforms that do not contain such intron. If the expressional odds of retention is bigger than 1.0, it means that the isoforms containing the RI are expressed more than the other isoforms, i.e., the expressional odds of retention is high. On the other hand, an expressional retention odds < 1.0 implies that the isoforms containing the RI are expressed less than the other isoforms with the RI spliced, i.e., the expressional odds of retention is low. The odds 1.0 suggests that the two types of isoforms are expressed at the same level.

We used the expressional odds of retention as criteria to form various subsets of RIs for the classification and distinction between RIs and CSIs. For example, the subset under “the 50/50 expressional odds of retention” stands for that we constructed a subset of RIs which have the 1.0 or larger expressional odds of retention, and used this subset of RIs to compare with the set of CSIs. Similarly, the subset under “the 10/90 expressional odds of retention” stands for that we constructed a subset of RIs which have the 1/9 or larger odds of retention, and used this subset of RIs to compare with CSIs. The default case is that we used machine learning methods to distinguish the whole set of RIs and the whole set of CSIs.

By the machine learning methods, the feature vector to represent the RIs and CSIs for the classification consists of the new feature FPKM and three types of conventional features (denoted as FeatureSet-1, FeatureSet-2, and FeatureSet-3). Given a RI, its FPKM value is the geometrical mean of the FPKMs of RI-containing isoforms in all the expressed samples. As a CSI is spliced out from all the isoforms of its gene, its FPKM is set as the geometrical mean of its FPKM values of the gene in all the samples. The three types of conventional features are similar to our previous study (Mao et al., 2014). The conventional FeatureSet-1 includes: the length of an intron, the nucleotide occurrence probabilities of A, C, G, and T, the AT content, the GC content and the segmental probabilities of four nucleotides correlation factors. The conventional FeatureSet-2 includes the signal strength features of the splice sites (SFvalue, SFaccvalue) and the similarity level features (IDdonv, IDacceptv). The conventional FeatureSet-3 includes frequent motifs features. These frequent motifs frequently occur in either the set of RIs or the set of CSIs but not in both. In order to identify these frequent motifs, one evaluation factor α(*x*(*k*)) is used to describe the diversity of a *k*-mer (from 2 to 5-mer) subsequence (*x*(*k*)) between the RIs and CSIs. A higher absolute value of α(*x*(*k*)) stands for a significant diversity of x(*k*) occurring in the RIs and CSIs. A negative value of α(x(*k*)) means a high frequency of *x*(*k*) in the RIs than in the CSIs, while a positive value of α(*x*(*k*)) signifies the opposite case. The other evaluation factor S(*x*(*k*)) means the confidence coefficients of *x*(*k*) in the RIs (S_*True*_ (*x*(*k*))) or CSIs (S_*False*_ (*x*(*k*))).

The classification is conducted using Random Forest (Nair et al., 2013), a machine learning algorithm. Measurements accuracy, F-Measure, and the area under a receiver operating characteristics curve (AUC) are used for the assessment of the classification performance. Definitions of these measurements can be referred to Liu et al. (2006).

## Results

### A large number of newly discovered RIs

**Figure 2:**
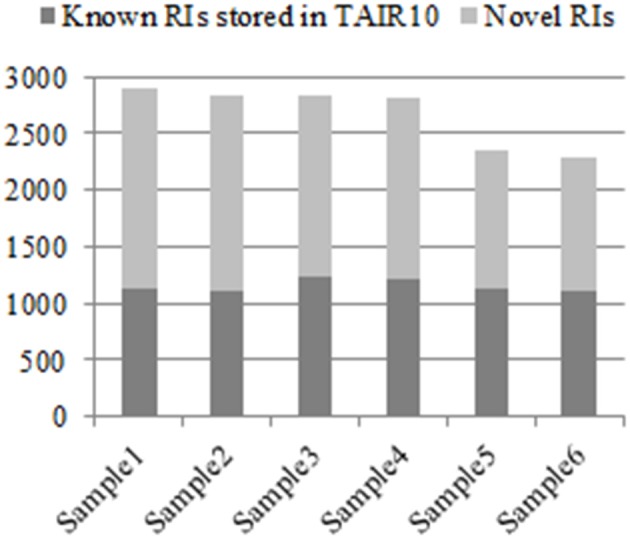
The known RIs in TAIR10 and novel discovered RIs respectively in six classes of RNA-Seq datasets. All experimental RNA-Seq datasets for analysis are divided into six classes: Sample1, Sample2, Sample3, Sample4, Sample5, and Sample6. For each class, that the discovered RIs are novel means they are unknown in TAIR10 but newly detected by our method, which depicted with light gray, and contrarily with dark gray.

From the six groups of RNA-Seq datasets Sample1, Sample2, Sample3, Sample4, Sample5, and Sample6, our method detected 2,904, 2,834, 2,834, 2,825, 2,346, and 2,298 RIs, respectively, (denoted as RI-set1, RI-set2, RI-set3, RI-set4, RI-set5, and RI-set6). The TAIR10 and the latest Araport11 Pre-release 3 (https://www.araport.org/data/araport11) have annotated 2,520 and 1,356 RIs, respectively, where the majority 1,289 RIs are common. Benchmarking with TAIR10, we identified 1,782, 1,719, 1,607, 1,603, 1,227, and 1,181 novel RIs, and re-confirmed 1,122, 1,115, 1,227, 1,222, 1,119, and 1,117 known RIs annotated in TAIR10 from Sample1, Sample2, Sample3, Sample4, Sample5, and Sample6, respectively (Figure 2).

**Figure 3:**
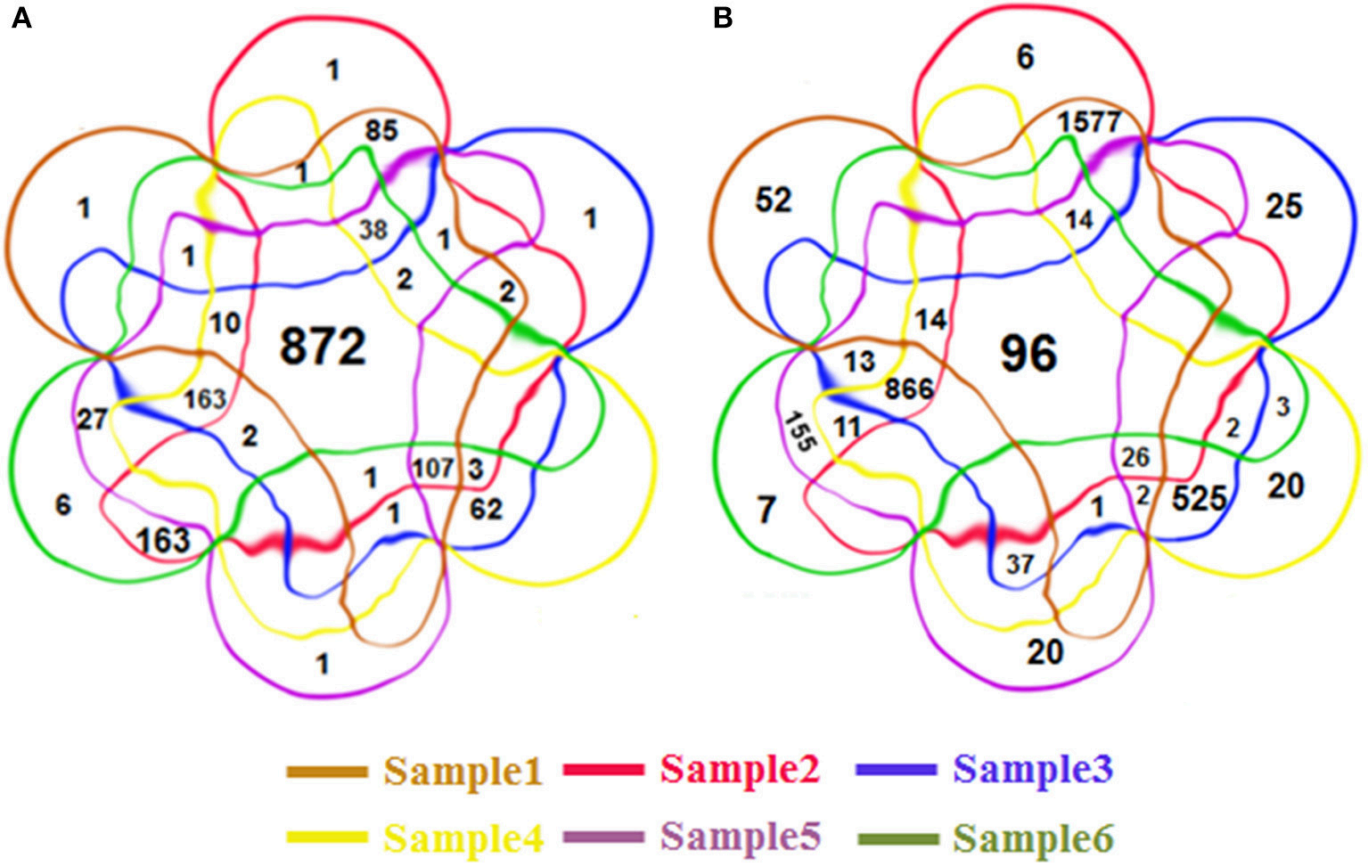
The 6-venn diagrams of RIs from six classes. The sets of RIs found from Sample1~6 have a large amount of overlap. **(A)** The known RIs unique to the six classes or their combinations. **(B)** The novel discovered RIs unique to the six classes or their combinations. They use the same color setting rules, red brown set represents Sample1, red set for Sample2, blue set for Sample3, yellow set for Sample4, purple set for Sample5, green set for Sample 6. The numbers of intersection areas reflect the details of overlap among the different classes.

Some RIs were detected repeatedly from these six groups of datasets. In total, we detected 4,856 distinct RIs, including 3,472 novel RIs and 1,384 RIs which have already been annotated in TAIR10. Figures 3A,B is 6-venn diagrams showing the numbers of RIs unique to these six datasets or their combinations. Some intersection areas have no number to fill, meaning there are no unique RIs for those overlapped areas of datasets.

The numbers of novel RIs unique to these datasets are listed in Table 2 in comparison with the numbers of known RIs annotated in TAIR10. The prefixes “RI-known” and “RI-novel” represent known RIs annotated in TAIR10 and novel RIs newly detected by our method. The suffix numbers in these notations indicate that the unique RIs belong to what datasets combinations. For example, RI-novel12 means the set of those novel RIs which had been detected from only Sample1 and Sample2 (i.e., AT1G55310, AT4G14300, AT1G28060 et al.).

**Table 2:** Intersections of known and novel RIs among six classes.

**Known in TAIR10**		**Novel**	
**Name of a RI set**	**Counts**	**Name of a RI set**	**Counts**
RI-known123456	872	RI-novel123456	96
RI-known12345	1	RI-novel13456	14
RI-known12356	2	RI-novel1234	26
RI-known13456	10	RI-novel1256	14
RI-known23456	2	RI-novel1345	1
RI-known1234	107	RI-novel3456	866
RI-known1235	1	RI-novel134	2
RI-known1256	38	RI-novel345	37
RI-known1345	1	RI-novel346	2
RI-known3456	163	RI-novel356	13
RI-known123	2	RI-novel456	11
RI-known124	1	RI-novel12	1,577
RI-known156	1	RI-novel34	525
RI-known234	3	RI-novel46	3
RI-known12	85	RI-novel56	155
RI-known34	62	RI-novel1	52
RI-known56	27	RI-novel2	6
RI-known1	1	RI-novel3	25
RI-known2	1	RI-novel4	20
RI-known3	1	RI-novel5	20
RI-known5	1	RI-novel6	7
RI-known6	2		

*The prefixes “RI-known” and “RI-novel” represent respectively known RIs in TAIR10 and novel RIs detected from RNA-Seq by our method. The suffix numbers show the unique RIs belong to serial numbers of datasets combinations*.

There are 872 of the 1,384 previously annotated RIs (63%) that are expressed in all of the six groups of datasets (Figure 3A). Of the 3,472 novel RIs, there are 96 RIs (2.76%) which are expressed in all of the six datasets (Figure 3B). These RIs (i.e., RI-known123456 and RI-novel123456) are combined, consisting of 968 RIs, for a special distinction with CSIs. This set of RIs is denoted as **RI-set-all-expressed**.

We are also interested in two specific subsets of the novel RIs (Figure 3B). One is the subset of 1,577 RIs which co-occurred only in the developmental tissues like seedlings, inflorescent meristems and flowers (namely, RI-novel12); the other is a subset of 866 RIs which co-occurred only under stress conditions like cold, heat, salt and drought (namely, RI-novel3456). These two RI sets are combined, denoted as **RI-set-stage-expressed**, for downstream analysis.

As defined, an intron is a CSI if and only if it is always spliced out from all the isoforms of one gene. A total of 73,048 CSIs were found from the six groups of RNA-Seq datasets. Fifty eight thousand four hundred and thirty six of them having a FPKM no < 0.5 were used to contrast with the RIs.

### Classification performance when the introns are represented by the new feature vector

Our newly discovered 4,856 RIs are much more than the 2,520 RIs currently annotated in TAIR10, and much more than the 1,356 RIs currently annotated in Araport11 Pre-release3. It is interesting to know whether the 4,856 RIs have distinct characteristics in comparison with the CSIs detected from the same six groups of RNA-Seq datasets.

As stated in the Method section, the feature vector of every intron consists of its FPKM value and its conventional features' values. FPKM describes the expression information of the intron at the isoform level. The definition is fixed for all the RI sets. The conventional FeatureSet-1 and FeatureSet-2 are also fixed and consistent across the eight sets to describe the introns. However, the conventional FeatureSet-3 are dynamically changed from RI-set1 to RI-set-stage-expressed depending on the values of the diversity factors and confidence coefficients for all the 2-5 mer motifs in each RI set (Table S1). All these feature vectors are shown in Table 3.

**Table 3:** The Feature vector to represent the RIs and CSIs for the classification.

**Feature types**	**Feature vector**
FeatureSet-1	Length; AT and GC content; nucleotide occurrence probabilities of A, C, G and T; Segmental probabilities of four nucleotides correlation factors (θ_*AG*_, θ_*AC*_, θ_*AT*_, θ_*GC*_, θ_*GT*_, θ_*CT*_).
FeatrueSet-2	SFvalue, SFaccvalue; IDdonv, IDacceptv
FeatureSet-3	**[RI-set1]—**“ACG,” “AGG,” “CCG,” “CGA,” “CGG,” “GAG,” “GCC,” “GGA,” “GGC,” “GGG,” “AAGC,” “AGAG,” “CAAG,” “GAAG,” “GAGA,” “GGAA,” “TATA,” “TTTT,” “AATTT,” “ATATT,” “ATTTT,” “TAATT,” “TATAT,” “TATTT,” “TTAAT,” “TTATA,” “TTATT,” “TTTTA“,“TTTTC“,“TTTTT”;
	**[RI-set2]—**“AGG,” “CCG,” “CGA,” “CGG,” “GAG,” “GCC,” “GGA,” “GGC,” “GGG,” “AAGC,” “AGAG,” “CAAG,” “GAAG,” “GAGA,” “GGAA,” “TATA,” “TTTT,” “AATTT,” “ATATT,” “ATTTA,” “ATTTT,” “TAATT,” “TATAT,” “TATTT,” “TTAAT,” “TTATA,” “TTATT“,“TTTTA“,“TTTTC“,“TTTTT”;
	**[RI-set3]—**“TCTTG,” “TCTCT,” “CTTTG,” “CTCTT,” “TTCTG,” “CATTT,” “TTTCT,” “AGG,” “CGA“,“GAAG“,“GGG“,“CCG“,“CGG”;
	**[RI-set4]—**“TCTTG,” “TCTCT,” “CTTTG,” “CTCTT,” “TTCTG,” “TTTCT,” “AGG,” “CGA,” “GAAG“,“GGG“,“CCG“,“CGG”;
	**[RI-set5]—**“CCG,” “CGA,” “CGG,” “GCG,” “GGG,” “AGGA,” “GAAG,” “TCGA,” “CATTT,” “CTTGT,” “TCTGT,” “TCTTG,” “TGCAG,” “TGCTT,” “TTGCA“,“TTGCT”;
	**[RI-set6]—**“CCG,” “CGA,” “CGC,” “CGG,” “GCG,” “GGG,” “AGGA,” “GAAG,” “TCGA,” “ACTTT,” “CATTT,” “TCTGT,” “TCTTG,” “TGCAG,” “TGCTT“,“TTGCT”;
	**[RI-set-stage-expressed]—**“CG,” “GG,” “CC,” “TT,” “TA,” “GAG,” “ACG,” “GGA,” “GCC,” “CGA,” “GGC,” “AGG,” “GGG,” “GCG,” “CCG,” “CGG,” “CGC,” “AAGC,” “GAGA,” “CAAG,” “GGAA,” “CAGA,” “GAAG,” “AGGA,” “TTTT,” “TATA,” “TTTTT,” “CTTTT,” “ATTTT,” “TTTGT,” “TGTTT,” “TTTTC,” “TTTTG,” “TTTCT,” “TCTTT,” “TTGTT,” “GTTTT,” “TTCTT”;
	**[RI-set-all-expressed]—**“CG,” “GG,” “ATA,” “CCG,” “CGG,” “TAT,” “TATA,” “ATAT,” “TAGT,” “TATT,” “CGGA,” “GGAG,” “GAGG,” “CAAG,” “ACCG,” “CGGT,” “TCGG,” “TATTT,” “CATTT,” “AATTT,” “ATTTT,” “TTTTA,” “TTATT,” “TTTTT,” “AAGAG,” “TGGAG”.
The Expression of Intron-containing Isoforms	**FPKM**
Class label	True (RIs); False (CSIs)

*The Feature vector consists of the expression of intron-containing isoforms (denoted as FPKM) and three types of conventional features (FeatureSet-1, FeatureSet-2, and FeatureSet-3). More definitions details of these conventional features can be referred to Mao et al. (2014)*.

**Figure 4:**
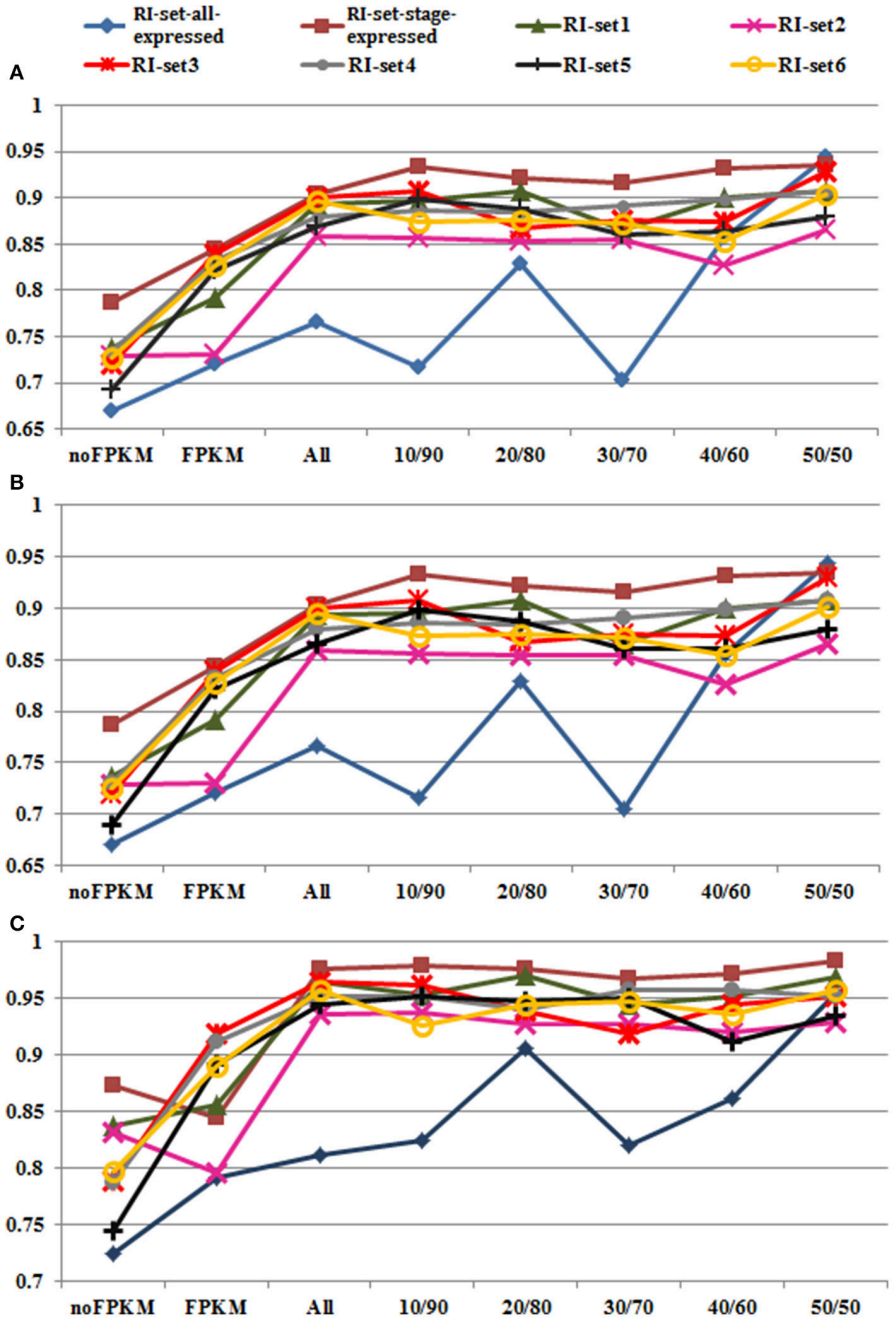
Performances of Random Forest in all datasets. Eight datasets (i.e., the green line with triangle marks for RI-set1, the pink line with 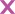 marks for RI-set2, the red line with ^
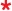
^ marks for RI-set3, the light gray line with filled circle marks for RI-set4, the black line with cross marks for RI-set5, the yellow line with hollow circle marks for RI-set6, the blue line with rhombus marks for RI-set-all-expressed, and the red brown with square marks for RI-set-stage-expressed) respectively represent the RIs and the corresponding CSIs extracted from Sample1, Sample2, Sample3, Sample4, Sample5, Sample6, all expressed in six datasets (RI-set-all-expressed) and co-occurred in the developmental tissues or under stress conditions (RI-set-stage-expressed). For each dataset, nofpkm (classification features except FPKM), fpkm (only FPKM feature) and various subsets of RIs (all classification features) depending on different criteria of expressional retention odds (all, 10/90, 20/80, 30/70, 40/60, and 50/50, Table S2) are prepared to do classification by Random Forest. **(A)** Depict the obtained performances of accuracy. **(B)** Describe the obtained performances of F-Measure. **(C)** Show the obtained performances of AUC. Obviously, 50:50 expressional odds of retention consistently reach the best overall performance (0.909 Accuracy and F-Measure, 0.954 AUC averagely) in all eight experimental datasets.

The classification and prediction were conducted through Random Forest (Weka 3.7). The 10-fold cross-validation performance by Random Forest was excellent on the 8 RI sets (i.e., RI-set1, RI-set2, RI-set3, RI-set4, RI-set5, RI-set6, RI-set-all-expressed, and RI-set-stage-expressed). Most of the AUC performance is around 95% (Table S2). The excellent performance is mainly attributed to the introduction of FPKM as a feature to characterize the introns. Without using this feature, the classification performance can drop 14.6% on accuracy, 14.7% on F-measure, and 14% on AUC as average, respectively, (Figure 4). These suggest that FPKM is an important feature to distinguish RIs from CSIs. The RIs usually have smaller values of FPKM (mean value = 15.2323, median = 5.3144) than CSIs (mean value = 25.2818, median = 8.7087), and the significant differences of FPKM between the RIs and CSIs were assessed using the one-way ANOVA (*P*-value < 2.2e-16). On the other hand, if only the FPKM feature is used to characterize the introns, the classification performance is not adequately good –FPKM has to be combined with the conventional sequence features for the excellent classification performance.

### 50/50 expressional odds of retention: perfect distinction between RIs and CSIs

**Figure 5:**
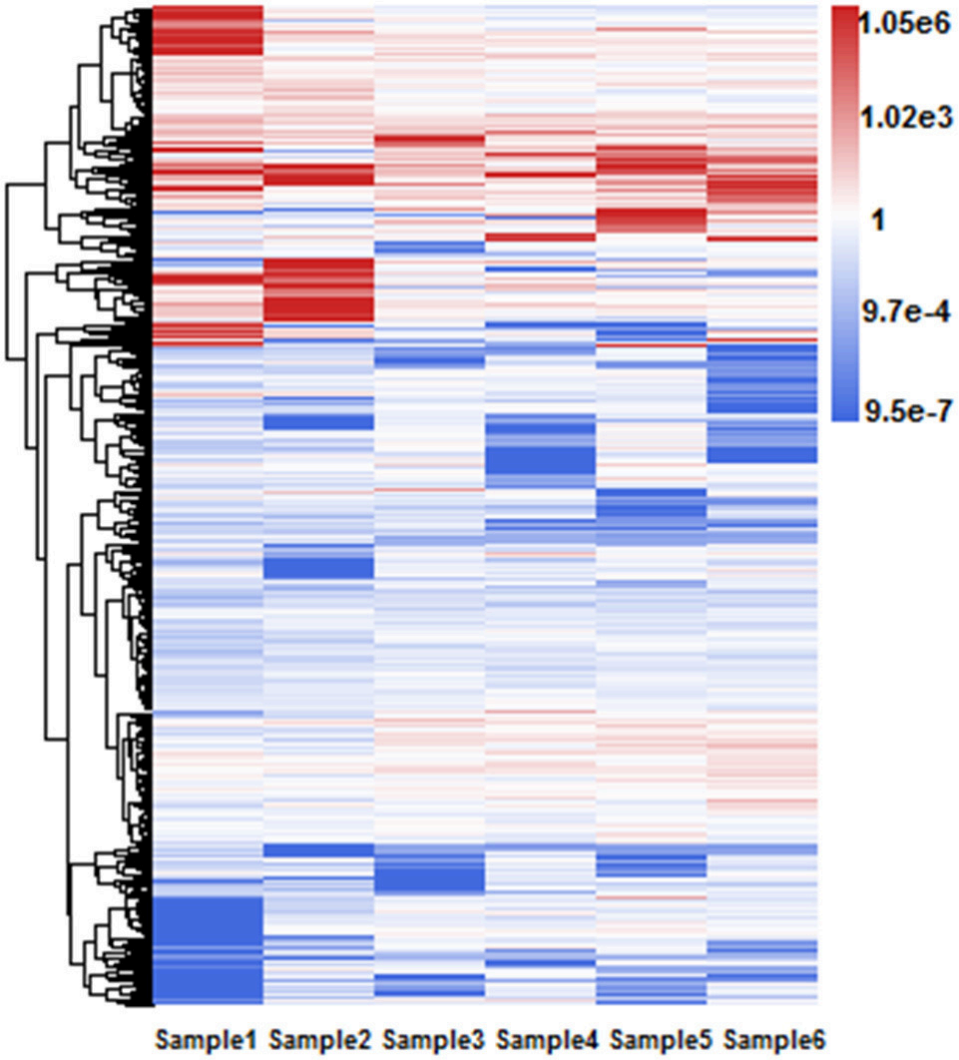
Hierarchical clustering of RI expressional retention odds for all RIs in RI-set-all-expressed. These 968 RIs of RI-set-all-expressed are expressed in all six classes. The blue area means that the expressional retention odds of these RIs < 1.0, while smaller value shows heavier blue color. The red area means the expressional retention odds of these RIs more than 1.0, while larger value shows heavier red color. The white area means that the expressional retention odds of these RIs are equal to 1.0.

Different RIs can have different expressional retention odds. Some RIs are highly retained in majority isoforms while the others are retained in only a few isoforms of one gene. For example, Figure 5 shows a hierarchical clustering analysis of RI expressional retention odds for all RIs of RI-set-all-expressed, where the red area means these RIs with more-than-1.0 expressional retention odds are easier to be retained in majority isoforms. Moreover, higher value of odds shows heavier red color. As typical representatives, the fourth intron of AT5G37370.4 is retained in three other majority isoforms (i.e., AT5G37370.1, AT5G37370.2, and AT5G37370.3). Similarly, the first intron of AT1G08570.2 is highly retained in three other isoforms (i.e., AT1G08570.1, AT1G08570.3, AT1G08570.4). On the contrary, the blue area means these RIs have a less-than-1.0 expressional retention odds. For example, the second intron of AT1G02090.1 and AT1G02090.2 is less retained in AT1G02090.3. We investigated whether the highly retained RIs can have a perfect distinction from the CSIs and what are their distinctive features.

To identify the best subset of RIs which can clearly distinguish from the CSIs, we constructed five subsets of RIs according to their expressional retention odds. For example, given RI-set1, the five subsets of RIs are: (i) the subset of RIs that have a expressional retention odds equal to or larger than 10/90, (ii) the subset of RIs that have a expressional retention odds equal to or larger than 20/80, (iii) the subset of RIs that have a expressional retention odds equal to or larger than 30/70, (iv) the subset of RIs that have a expressional retention odds equal to or larger than 40/60, and (v) the subset of RIs that have a expressional retention odds equal to or larger than 50/50. The suffixes “g10,” “g20,” “g30,” “g40,” and “g50” represent the expressional retention odds 10/90, 20/80, 30/70, 40/60, and 50/50, respectively, (Table S2).

The Random Forest classification performances and the optimal parameters on the all RI subsets are shown in Table S2. The changing trends of Accuracy, F-Measure and AUC on the eight RI sets are depicted in Figures 4A–C respectively. On the whole, Accuracy and F-Measure have similar tendencies because of no more than 0.2% differences between them from 10/90 odds to 50/50 odds in the eight RI sets. In Figure 4C, the performances of AUC appear to be better than those of Accuracy and F-Measure in all RI sets. Moreover, we discovered that performance enhancements in Accuracy, F-Measure and AUC were unstable from 10/90 to 40/60 compared with the eight default RIs sets. For instance, with the exception of subtle differences in AUC performance, some datasets (RI-set1, RI-set4, and RI-set-stage-expressed) indeed obtained increases of classification performance, some datasets (RI-set2) were in the opposite direction, the reminder of datasets (RI-set3, RI-set5, RI-set6, and RI-set-all-expressed) appeared to a bit up and down. However, 50/50 or larger expressional odds of retention consistently reached the best overall performance (i.e., 0.909 Accuracy and F-Measure, 0.954 AUC average) in all sets.

### Length of introns, GC content, distribution of CDS, and features of splice sites

We focus on a case study to understand characteristics of RIs in RI-set-stage-expressed. The comparison with the whole set of CSIs and with a subset of RI-set-stage-expressed (those RIs of more-than-1.0 expressional retention odds, namely RI-set-stage-expressed_g50, or RIg50 for short). The intron length distributions of these three sets of introns are presented in Table 4. The introns in RIg50 have the widest length range and the biggest mean value. Moreover, the GC content of the RIs in RIg50 is highest (41.5%), followed by the RIs in RI-set-stage-expressed (35.91%), and the lowest was found in the set of CSIs (32.66%).

**Table 4:** The GC content, length distribution quartiles of introns and the mean values of splice regulating factors in CSIs, RIs, and RIg50.

**Dataset**	**GC (%)**	**SFvalue**	**SFaccvalue**	**IDdonv**	**IDacceptv**	**Length**	**Length distribution of introns [minimum, 0.25, 0.5, 0.75, maximum]**
		**[mean values]**					
CSIs	32.66	4.8691	6.4489	18.453	18.362	155	[20, 86, 99, 156, 155]
RIs	35.91	4.1038	5.0915	18.221	18.056	140	[15, 82, 96, 134, 213]
RIg50	41.15	1.8208	2.1530	17.788	18	260	[18, 84, 119, 266, 522]

*Our focus is the differences of them among CSIs, RIs, and RIs of more-than-1.0 expressional retention odds of RI-set-stage-expressed (namely RIg50). In the following tables, CSIs, RIs and RIg50 are defined as the same. SFvalue and SFaccvalue represent the strength of splice sites. IDdonv and IDacceptv represent the similarity between two flanking sequences of splicesites*.

For the splice sites, there were only 53.79% of RIg50 that have the consensus GT-AG introns, much lower than that of the CSIs (99%) and the RIs in RI-set-stage-expressed (96.7%). There were a higher proportion of RIs in RIg50 (73.90%) than those in RI-set-stage-expressed (61.36%) occurred in the CDS region (Table 5). Such RIs found in the CDS region have a greater chance to be translated into proteins.

**Table 5:** The occurrence of RIs and RIg50 in CDS or UTR, according to TAIR10.

**Dataset**	**CDS**	**CDS+UTR**	**UTR**	**Proportion**
RIs	594	42	332	61.36%
RIg50	507	78	101	73.90%

**Figure 6:**
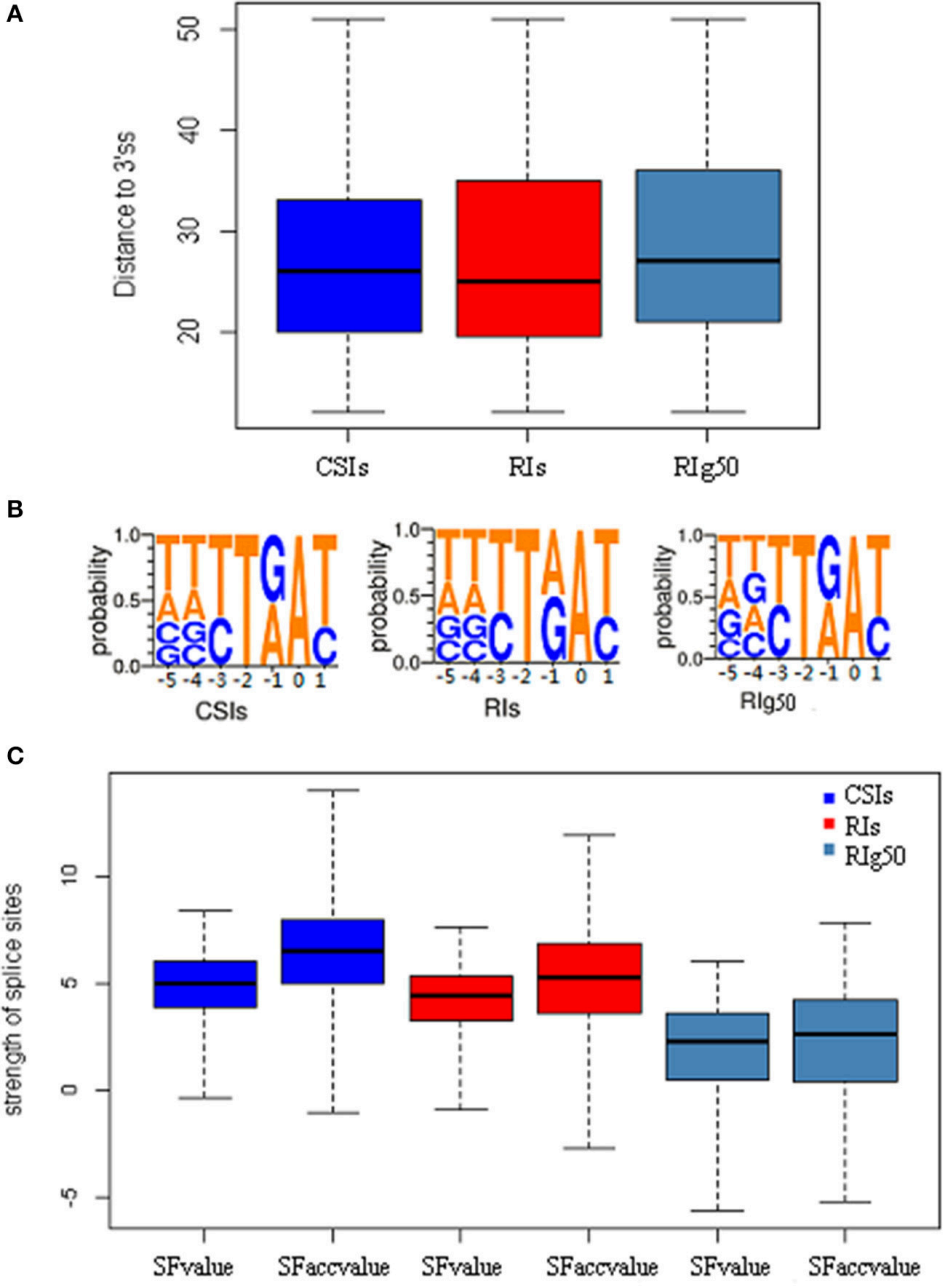
The distribution features of conservative sequence motifs at the branch point. Here all negative samples (CSIs, blue color), positive samples(RIs, red color) and positive samples with more-than-1.0 expressional retention odds of RI-set-stage-expressed (RIg50, navy blue color) are chosen for the comparisons. **(A)** The length distribution of branch point sequence motifs from the acceptor site (3′ss) to the branch point A. **(B)** The weblogo of the branch point sequence motifs. The vertical scale indicates the nucleotide occurrence probabilities of A, T, C, and G. All branch point sequence motifs is strictly aligned with the branch point (A, 0 point). **(C)** The features of splicesites. SFvalue, SFaccvalue indicate the strength of the splice sites, and IDdonv, IDacceptv represent the similarity between two flanking sequences of corresponding splice sites.

We also studied the strength of splice sites (namely, SFvalue and SFaccvalue) and the similarity measurements between the two flanking sequences of splice sites (namely, IDdonv and IDacceptv) for the RIs in RIg50 and RI-set-stage-expressed in comparison with the CSIs. We found that the RIs in RIg50 have the smallest SFvalue, SFaccvalue, IDdonv, and IDacceptv (Figure 6C and Table 4). The maximum relevance minimum redundancy (mRMR) method (Peng et al., 2005) was applied to these intron sets and selected SFaccvalue and SFvalue as top2 and top4 classification features (File S3). All these indicate that the introns in RIg50 have subtle and special strength of splice sites that makes the retention easier than those in RI-set-stage-expressed.

### Conservative sequence motifs at the branch point

The branch point sequence of a splice site is located at the upstream of the polypyrimidine tract. The binding of U2-snRNP to the branch point A has a strong influence on the splicing result (Zhang et al., 2011). Xia et al. reported that the average distance between the branch point and the acceptor site was 33–34 bp, according to 19 experimentally proven branch point sequences (Xia et al., 2006). Marquez et al. reported a conservative motif YTRAY which was frequent for U2 introns in Arabidopsis (Marquez et al., 2012). In yeast, the branch point sequence is TACTAAC and is almost invariant (Berglund et al., 1997). Mercer et al. discovered a set of 5- to 7-nt branch point sequence motifs via a genome-wide identification of 59,359 high-confidence human branch point (Mercer et al., 2015).

We examined a conservative motif NNYTRAY near the acceptor sites in terms of position and structure features of branch point sequences. By our study, all the introns longer than 58 bp length were strictly aligned with the branch point A (0 point), where NNYTRAY (−5~+1 bp) was searched within a distance between 11 and 52 bp in the upstream of the acceptor site (3′ss). The occurrences of the conservative motif NNYTRAY are shown in Table 6. The occurrence rate of NNYTRAY in the CSIs is the highest (59.8%), followed by the occurrence rate for the RIs in RI-set-stage-expressed (48.5%) and that for the RIs in RIg50 (only 34.1%). Figure 6A describes the length distributions from 3'ss to branch point A for the CSIs and RIs in RI-set-stage-expressed and RIg50. The minimum and maximum distance between the branch point A and the 3′ss are consistent in the three intron sets, only 0.25, 0.5 and 0.75 quantiles of distance between the branch point A and the 3'ss show subtle differences (within −1~ + 1 bp range, Table 6).

**Table 6:** The occurrence of the conservative motif NNYTRAY in CSIs, RIs and RIg50.

**Dataset**	**Length of intron < 58 bp**	**Length of intron ≥ 58 bp**		**Distribution of distance to the accepter site [minimum,0.25, 0.5, 0.75, maximum]**
		**Introns Number without NNYTRAY**	**Occurrence Numbers of NNYTRAY**	
CSIs	1	811	1188	[12, 20, 26, 33, 51]
RIs	29	420	423	[12, 19.5, 25, 35, 51]
RIg50	52	400	234	[12, 21, 27, 36, 51]

*Distribution of distance to the accepter site (3′ss) respectively illustrate the minimum, 0.25, 0.5, 0.75 quantiles, and the maximum distance between the branch point A and the 3′ss*.

The detected structures of NNYTRAY are shown in Figure 6B. It is clear that subsequence (−3~+ 1 bp) have higher conservation while nucleotides at −4 and −5 positions were not conserved (may be A, T, G or C) for CSIs and RIs in RI-set-stage-expressed and RIg50. The occurrence frequency of G is second to T in RIg50, unlike the other two intron sets where the second nucleotide is A. The frequency details of A, T, C, and G in each position (−5~+1) in CSIs, RI-set-stage-expressed and RIg50 are shown in Table 7. The nucleotides at −3 and +1 positions are similar while nucleotide at −1 position has subtle difference.

**Table 7:** The frequency details of A, T, C, and G in each position of the conservative motif NNYTRAY in CSIs, RIs, and RIg50.

**Classes**	**Nucleotide**	**−5**	**−4**	**−3**	**−1**	**1**
CSIs	A	0.227273	0.251684	0	0.476431	0
	C	0.175926	0.164983	0.367845	0	0.298822
	G	0.157407	0.175084	0	0.523569	0
	T	0.439394	0.408249	0.632155	0	0.701178
RIs	A	0.247863	0.226496	0	0.465812	0
	C	0.15812	0.209402	0.435897	0	0.337607
	G	0.239316	0.260684	0	0.534188	0
	T	0.354701	0.303419	0.564103	0	0.662393
RIg50	A	0.247863	0.226496	0	0.465812	0
	C	0.15812	0.209402	0.435897	0	0.337607
	G	0.239316	0.260684	0	0.534188	0
	T	0.354701	0.303419	0.564103	0	0.662393

*For −2 and 0 point, T and A are respectively constant nucleotides*.

It is suggested that the conservative motif of branch point sequence YTRAY in Arabidopsis has been mainly distributed in the range of 20–36 bp distance to 3′ss. We also found that the introns without YTRAY near 3′ss had a great possibility to be retained.

### Contrasting motifs between RIs and CSIs themselves

As show in Table 3, the conventional FeatureSet-3 (frequent motifs features) are different among these RI sets based on the results of diversities (α(*x*(*k*))) and confidence coefficients (S_*True*_ (*x*(*k*)) and S_*False*_ (*x*(*k*))) of all 2 to 5-mer motifs (see Table S1). Some typical frequent motifs were selected via suitable thresholds of diversities and confidence coefficients for each individual RIs set. We focused on a case study to understand the influence of frequent motifs features by the comparison with the RI-set-all-expressed and RIg50. Table S3 shows values of α(x(k)), S_*True*_ (*x*(*k*)) and S_False_ (*x*(k)) of some typical frequent motifs in the RI-set-all-expressed while Table S4 shows those in the RIg50. The outstanding differences for thresholds of α(x(k)) and S(*x*(*k*)) between the two RIs sets were discovered. In the first one, the diversities and confidence coefficients of frequent motifs were incompatible, so a compromise of thresholds are set as (α(x(k)) < −0.36 and S_*True*_ (*x*(*k*))> 0.11) or α(x(k)) > 0.22 and S_False_ (*x*(k))> 0.35). While in the second one, these two indexes were in balance and the relatively high thresholds of them were set as (α(x(k)) < −0.55 and S_*True*_ (*x*(*k*))> 0.45) or α(x(k)) > 0.45 and S_False_ (*x*(k)) > 0.5). Obviously, the classification performances in RIg50 are improved significantly by comparison to RI-set-all-expressed (AUC: 0.983 vs. 0.812). It suggests that these distinguishable frequent motifs are easier to be found by comparison between the CSIs and the RIs with more-than-1.0 expressional retention odds.

As typical representatives, “GGG-containing,” “GGAG-containing,” “AT/TA-rich,” “AG/GA-rich,” and “TTTT-containing” frequent motifs are investigated. In RI-set-all-expressed, the values of α(*x*(GGG − containing motifs)), α(*x*(GGAG − containing motifs)) and α(*x*(AG/GA − rich motifs)) are less than zero (−0.36, −0.55, and −0.27), which indicates that these motifs appear frequently in the RIs than CSIs. While the mean values of α(*x*(AT/TA − rich motifs)) and α(*x*(TTTT − containing motifs)) are greater than zero (0.25 and 0.19), which illustrates the opposite case. The similar results are obtained in the RIg50, but the absolute values of diversities and confidence coefficients of these frequent motifs are much higher in RIg50 than RI-set-all-expressed (Table 8). All these indicate that the selected frequent motifs have contributed to better distinction between RIs and CSIs in RIg50.

**Table 8:** The assessment indexes of typical representatives for the conventional FeatureSet-3 in RIg50.

**Motifs**	**α(*x*(*k*))**	***S*_*True*_ (*x*(*k*))**	***S*_*False*_ (*x*(*k*))**
	**Mean values**		
GGG-containing	−0.77	0.14	
GGAG-containing	−0.92	0.21	
**AG/GA-rich(4bp)**	−**0.72**	**0.50**	
TA/AT-rich(4–5bp)	0.33		0.28
**TTTT-containing**	**0.54**		**0.57**

*α(x(k)) indicates the diversities of x(k) (typical frequent motifs) between in RIg50 and CSIs. S_True_(x(k)) indicates the confidence coefficients of x(k) in RIg50, and S_False_(x(k)) indicates the same in CSIs*.

*The bold “AG/GA-rich” and “TTTT-containing” motifs are putative intronic splicing silencers (ISSs) and intronic splicing enhancers (ISEs)*.

### Pathways involving the genes which contain co-occurring RIs in multiple samples

The RIs in RI-set-all-expressed are those RIs, which are expressed in all of the six RNA-Seq datasets (namely, Sample1, Sample2, Sample3, Sample4, Sample5, and Sample6). Similarly, the RIs in RI-set-stage-expressed are those RIs which are expressed in Sample1 and Sample2 only, or expressed in Sample3, Sample4, Sample5, and Sample6 only. These RIs are called co-occurring RIs in multiple samples. The two subsets of RI-set-stage-expressed and RI-set-all-expressed that have an expressional retention odds equal to or larger than 50/50 (RIg50 and RI-set-all-expressd_g50) can be clearly separated from the CSIs by Random Forest (Accuracy = 0.935, F-Measure = 0.935 and AUC = 0.983 for the first one; Accuracy = 0.944, F-Measure = 0.944 and AUC = 0.955 for the second one; see Table S2). We hypothesize that these co-occurring RIs with more-than-1.0 expressional retention odds could perform the global regulation of alternative splicing.

**Figure 7:**
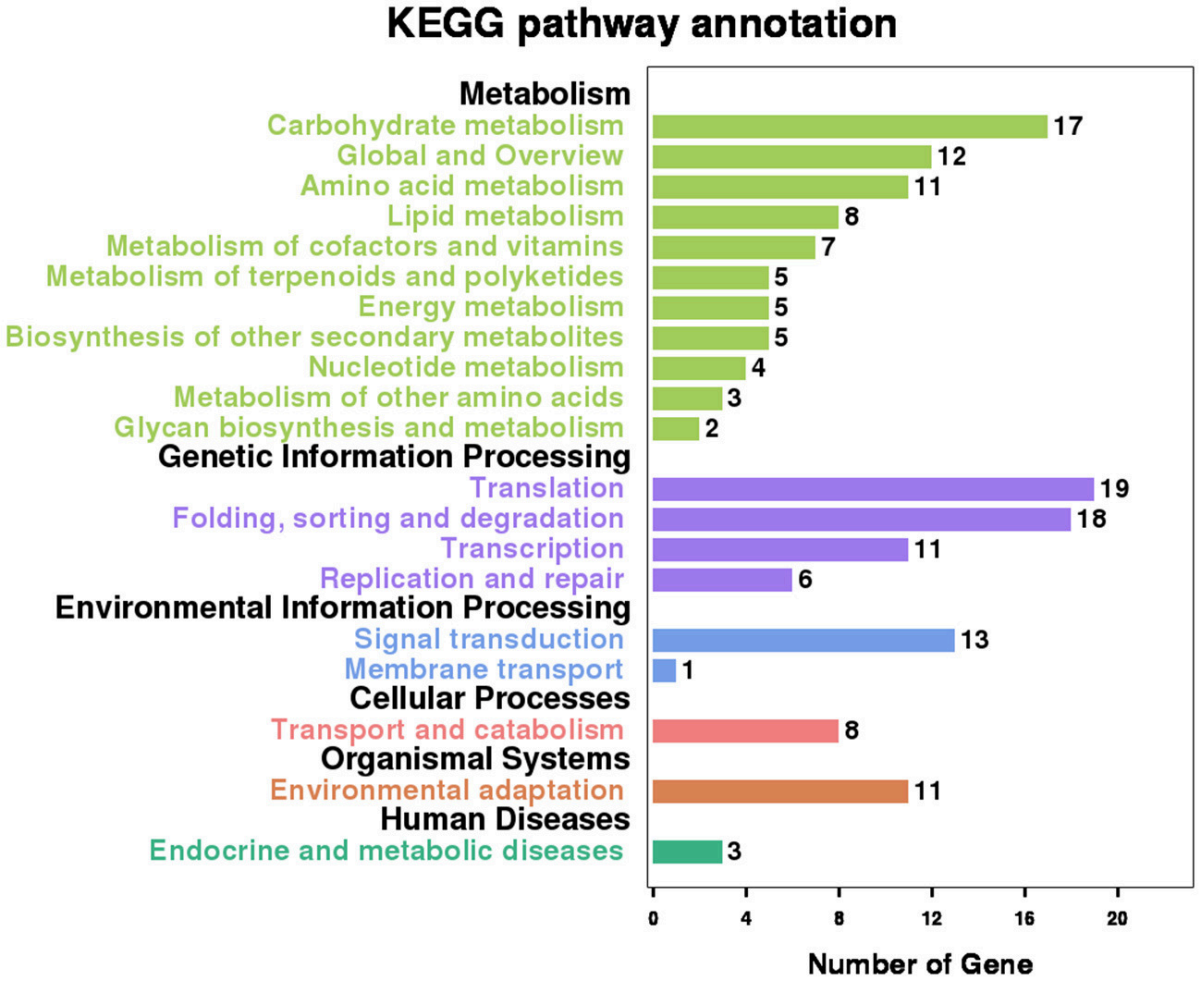
Significant KEGG enrichment pathway involving the genes which contain co-occurring RIs in multiple Samples.

There are 556 genes containing these co-occurring RIs. Pathway analysis with the Kyoto Encyclopedia of Genes and Genomes (KEGG) database shows that 109 (19.6%) genes have significant enrichment in 20 main categories that cover 79 pathways (Figure 7 and Table S5). “Spliceosome” and “mRNA surveillance pathway” are the two typical and representative pathways related to regulation of alternative splicing. For examples, an intron of ATSRL1 (AT5G37370; File S4) was identified alternatively retained in some isoforms for all the six samples (expressional retention odds reaching to the level of 12.24, 87.54, 16.03, 13.68, 6.72, and 9.6 in the six samples, respectively), which would generate truncated Serine/Arginine-Rich (SR) like protein isoforms; As members of the SR protein and hnRNPs (heterogeneous nuclear ribonucleopartile proteins) families, ATSCL33 (AT1G55310), and DL3190W (AT4G14300; File S4) harbored intron retention events from RNA-Seq analysis in Sample1 and Sample2; Meyer et al. reported that mutants defective in RNA-Binding proteins implicated in the splicing process (Meyer et al., 2015).

## Discussion

There are several methods (e.g., MISO, rMATS, and SpliceGrapher) that have been developed to detect patterns of alternative splicing (including intron retention patterns) using RNA-Seq data (Katz et al., 2010; Rogers et al., 2012; Shen S. et al., 2014). However, these tools still have limits for biologists to have accurate identification of RIs and CSIs. MISO is limited to only discovering isoforms because of its heavy reliance on the annotations of known alternative events; rMATS is unable to detect differential alternative splicing patterns between two RNA-Seq datasets without a user-defined threshold; SpliceGrapher cannot quantify differential isoforms expression, although it can predict some novel alternative splicing events at the splice graph level. Therefore, these tools cannot meet the expectations and needs for more comprehensive and accurate annotation of RIs and CSIs using RNA-Seq data. On the other hand, some previous reports are related with alternative splicing in Arabidopsis. Filichkin et al. has identified stress-associated alternative splicing events, and 40 percent of them are intron retention events (Filichkin et al., 2010). Marquez et al. utilize directly ASTALAVISTA software for their identification of RIs, which had lead to plenty of redundant records (Marquez et al., 2012). Wang et al. studied the patterns of alternative splicing in flower development stages, and found a higher proportion of RI (54.8%) in all alternative splicing events (identified from 25.6% of TAIR10 annotated genes; Wang et al., 2014). However, they usually focus on one single stress-, tissue- or growth stage RNA-Seq source, and the research results of alternative splicing are far from comprehensive and accurate in Arabidopsis. Given the increasing availability of public RNA-Seq datasets, there is a high demand to have a capable method that can: (i) detect RIs and CSIs accurately, (ii) perform the expression quantification at the isoform level, and (iii) benchmark against the existing annotation database of Arabidopsis. So in this study, we present a computational pipeline including quality control metrics, transcript alignment and reconstruction, express quantification, redundancy identification, and integrated analysis on multiple RNA-Seq data. Our pipeline is extensible for processing more RNA-Seq data and can be modified to study other plant species. In this study, we detected 4,856 RIs from18 RNA-Seq datasets using our pipeline. In addition, commonality and diversity of these RIs in different datasets were analyzed. There are 986 RIs that were expressed in all 6 sample sets. With comparison of TAIR10, 3,472 novel RIs were evident in our data analysis of 18 RNA-Seq datasets, which allowed us to examine 58,346 CSIs accurately. All these indicate that our method can facilitate accurate and comprehensive identification of RIs and CSIs in Arabidopsis, as well as in other plant species.

Previous studies usually use conventional sequence features to predict alternative splicing events on a genome scale (Jian et al., 2014; Mao et al., 2014). We investigated the quantitative expression information of intron-containing isoforms (FPKM), and discovered significant differences of FPKM occurring between RIs and CSIs (15.2323 vs. 25.2828, averagely). It is interesting to note that the FPKM feature ranks in the top3 when we evaluate our selected features of classification through mRMR method, the “ATTTT” and SFaccvalue features sort in the top1 and top2 respectively (File S3). Meanwhile FPKM feature proved to dramatically contribute in distinguishing RIs from CSIs by our experiments. Furthermore, we also studied whether the highly retained RIs can have a perfect distinction from the CSIs. We researched five subsets of RIs according to their expressional retention odds (from 10/90 to 50/50), and found that 50/50 expressional odds of retention consistently gained the outstanding performance of AUC 0.95 on average in all datasets. In addition, some conventional features related with alternative splicing were surveyed among representative RIs with more-than-1.0 expressional retention odds (RIg50). It is noted that GC content (41.5 vs. 35.91%) and the occurrence rate in CDS region (73.90 vs. 61.36%) are obviously higher in RIg50 than RIs, while the strength of splice sites (SFvalue, 1.8208 vs. 4.1038; SFaccvalue, 2.1530 vs. 5.0915), the similarity between two flanking sequences of splice sites (IDdonv, 17.788 vs. 18.221; IDacceptv, 18 vs. 18.056) and the consensus GT-AG introns (53.79 vs. 96.7%) appear lower in RIg50 than RIs. It is likely that RIs with higher expressional retention odds have shown more stronger signal strength of retention than those with low expressional retention odds.

RIs is the predominant event of alternative splicing in plants (Meyer et al., 2015; Staiger, 2015). *A. thaliana*, as an important model plant with abundant genetic resources, has greatly advanced our knowledge of the recognition mechanism of RIs in plants. Our study found that RIs have distinguishable features in comparison with CSIs by Random Forest, especially when RIs tend to express in multiple samples and possess higher expressional retention odds (more-than-1.0). Based on the co-occurring RIs, we researched these distinguishable features in more details. In addition to the low strength of splice sites and high similarity with the flanking exon sequences, low occurrence percentage of YTRAY near the acceptor site, putative ISSs (AG/GA-rich motifs) and ISEs (TTTT-containing motifs) are closely related to the recognition mechanism of RIs. On the other hand, we also performed KEGG pathway analysis in 556 genes containing the co-occurring RIs. The results show significant enrichment in 79 pathways, especially the enrichment of RNA-Binding proteins. The RIs of ATSRL1, ATSCL33, and DL3190W can illustrate the regulation in the splicing process. All these indicate that the distinguishable features reflected obviously in co-occurring RIs should play a wide range of functions in alternative splicing process, which is independent of tissue-, growth-stage or stress-specific environment.

It has been previously reported that the regulatory elements “GAAG” within the introns of ATSCL33 can imply an auto-regulation of alternative splicing (Thomas et al., 2012; Meyer et al., 2015). In our study, the mean value of diversities α(*x*(AG/GA − rich motifs)) between the RIg50 and the CSIs is −0.72, while the confidence coefficents in the RIg50 (S_*True*_(*x*(AG/GA − rich motifs))) is 0.50 (much larger than (S_*True*_(*x*(GGG − containing motifs)), 0.14; S_*True*_(*x*(GGAG − containing motifs)), 0.21) (See Table 8). These results indicate that AG/GA-rich motifs, such as, “AGGA,” “GAAG,” “AGAG,” and “GAGA,” have occurred more frequently in the RIg50 than in the CSIs. They should have played a role of intronic splicing silences (ISSs) in Arabidopsis. We found that there are multiple AG/GA rich motifs located in two introns of ATSCL33. These AG/GA-rich motifs involved in regulating intron retention in isoforms of ATSCL33. In DL3190 W and ATSRL1, we discovered the same regulation function of AG/GA-rich motifs within the corresponding RIs in pre-mRNA splicing. Accordingly, TTTT-containing motifs held higher mean values of both α(*x*(TTTT − containing motifs)) (0.54) and S_*False*_(*x*(TTTT − containing motifs)) (0.57) than those of TA/AT-rich motifs (0.33 and 0.28), which are putative intronic splicing enhancers (ISEs) as suggested by a previous study (Mao et al., 2014). So, TTTT-containing motifs, instead of TA/AT-rich motifs, seem to be the ISEs because of more obviously abundance in the CSIs.

RDM16 (AT1G28060) regulates the formation of the U4/U6-associated splicing factor. Huang et al. illustrated 308 intron retention events discovered in RDM16 by RNA-Seq analysis (Huang et al., 2013). Although RDM16 did not occur alternative splicing under stress conditions (Sample3–Sample6) and its alternative splice event is not annotated in TAIR10 database, but TCONS_00002758 and TCONS_00002760 of RDM16 in our study were clearly detected in developmental tissues (Sample1 and Sample2). The second intron of TCONS_00002758 were proved in retention within the second exon of TCONS_00002760, confirming that RDM16 had an impact on the pre-mRNA splicing in Arabidopsis. ABH1 (AT2G13540) encodes a nuclear cap-binding protein that is involved in ABA signaling and flowering. Our analysis indicates that some specific RIs with more-than-1.0 expressional retention odds were discovered in ABH1 (File S4). The results highlight that the genes involving the co-occurring RIs in multi-samples are closely related to pre-mRNA splicing in Arabidopsis.

However, some of RIs show inconsistency with significant difference of expression in different environmental treatments or developmental stages. To identify RIs' regulation in response to different conditions, it is challenging to eliminate systematic variations among RNA-Seq datasets with different platforms or from different labs. An effective way to describe the expression correlations in different datasets is to construct co-splicing networks (Li W. et al., 2014; Klepikova et al., 2016), which will increase the stability and spatiotemporal specificity of gene expression profiles. On the other hand, the latest PacBio long-read sequencing can help us validate alternative splicing isoforms assembled from short reads systematically (Li et al., 2016). These will be conducive to understanding of regulation mechanisms involved in spatiotemporal specific RIs.

## Author contributions

Experimental design: JL and RM; Experiments implementation: RM; Data analysis: RM; Manuscript preparation: RM, CL, YZ, and XH; Supervision: JL and CL; Funding: RM and JL.

### Conflict of interest statement

The authors declare that the research was conducted in the absence of any commercial or financial relationships that could be construed as a potential conflict of interest.
