# Supplementary material for: 50/50 Expressional Odds of Retention Signifies the Distinction between Retained Introns and Constitutively Spliced Introns in Arabidopsis thaliana
Source: Front Plant Sci. 2017 Oct 9;8:1728. doi: 10.3389/fpls.2017.01728 (PMC5640774; doi:10.3389/fpls.2017.01728)
Supplement: Supplementary file 3 [file Presentation1.PDF]

Supplementary Material

Article Title

50/50 Expressional Odds of Retention Signifies the Distinction between Retained Introns and Constitutively Spliced Introns in *Arabidopsis thaliana*

Rui Mao[1*], Chun Liang[2,3], Yang Zhang[1], Xingan Hao[4] and Jinyan Li[5,*]

[1]College of Information Engineering, Northwest A&F University, Yangling, Shaanxi 712100, China.

[2]Department of Biology, Miami University, Oxford, Ohio 45056, USA.

[3]Department of Computer Sciences and Software Engineering, Miami University, Oxford, Ohio 45056, USA.

[4]State Key Laboratory of Crop Stress Biology for Arid Areas, College of Plant Protection, Northwest A&F University, Yangling, Shaanxi 712100, China.

[5]Advanced Analytics Institute, University of Technology Sydney, 81 Broadway, NSW 2007, Australia.

*** Correspondence**

RM: maorui@nwafu.edu.cn

CL: liangc@miamioh.edu

YZ: zhangyang@nwafu.edu.cn

XH: haoxingan@nwafu.edu.cn

JL: jinyan.li@uts.edu.au

1 Supplementary Data

File S1. All extracted data used in the article. (.zip, 40.7MB, please download from http://pan.baidu.com/s/1slwGIYh)

File S2. The user manual and open-source of RIs_CSIs_ID. (.zip)

File S3. The order of performance for classification features based on the maximum relevance minimum redundancy (mRMR) method. (.xlsx)

File S4. Some representative genes, which contain co-occurring RIs in multiple Samples that are closely related to pre-mRNA splicing in Arabidopsis. (.pdf)

2 Supplementary Tables

Table S1 (TableS1.xls). The values of evaluation indexes (($\alpha(x(k))$ and $S(\boldsymbol{x}(k))$) of all k-mer (from 2 to 5-mer) motifs for all eight datasets.The $\alpha(x(k))$ is used to describe the diversity of a k-mer subsequence ($x(k)$) between the RIs and CSIs, and the $S(x(k))$ means the confidence coefficients of $x(k)$ in the RIs ($S_{True}(x(k))$) or CSIs ($S_{False}(x(k))$). The eight datasets are named as RI-set1, RI-set2, RI-set3, RI-set4, RI-set5, RI-set6, RI-set-all-expressed, and RI-set-stage-expressed. They respectively represent the source of RIs extracted from Sample1, Sample2, Sample3, Sample4, Sample5, Sample6, all expressed in six datasets and co-occurring in the developmental tissues or under stress conditions. For each dataset, the conventional FeatureSet-3 are selected through the above values of evaluation indexes.

Table S2. Optimal parameters and performances of Random Forest in all datasets. The prefix codes of RI-set1, RI-set2, RI-set3, RI-set4, RI-set5, RI-set6, RI-set-all-expressed, and RI-set-stage-expressed respectively represent the same meaning with the Table S1. The Suffix codes of nofpkm and fpkm stand for classification features except FPKM or only FPKM feature. The other datasets without these two suffixes utilise all classification features to work. The suffixes “g10”, “g20”, “g30”, “g40” and “g50” correspondingly represent RIs with 10/90, 20/80, 30/70, 40/60 and 50/50 expressional retention odds.

**Data set**	**Parameters**		**Performance of random forest**			**Positive samples**	**Negative samples (CSIs)**	**Total samples (RIs)**
	**numFeatures**	**numTrees**	**Accuracy**	**F-Measure**	**AUC**			
RI-set1_nofpkm	7	69	0.737	0.737	0.837	2904	3000	5904
RI-set1_fpkm	1	70	0.792	0.792	0.856	2904	3000	5904
RI-set1	7	70	0.893	0.893	0.964	2904	3000	5904
RI-set1_g10	8	63	0.896	0.895	0.953	1933	1900	3833
RI-set1_g20	8	43	0.908	0.908	0.97	1682	1700	3382
RI-set1_g30	7	34	0.866	0.866	0.945	1492	1500	2992
RI-set1_g40	7	33	0.9	0.9	0.951	1356	1350	2706
**RI-set1_g50**	**7**	**25**	**0.908**	**0.908**	**0.969**	**1202**	**1200**	**2402**
RI-set2_nofpkm	6	68	0.729	0.728	0.832	2834	3000	5834
RI-set2_fpkm	1	68	0.731	0.731	0.796	2834	3000	5834
RI-set2	6	68	0.859	0.859	0.936	2834	3000	5834
RI-set2_g10	8	60	0.857	0.856	0.937	2095	2100	4195
RI-set2_g20	8	58	0.854	0.855	0.928	1770	1800	3570
RI-set2_g30	7	42	0.855	0.854	0.927	1572	1600	3172
RI-set2_g40	7	35	0.827	0.826	0.92	1385	1400	2785
**RI-set2_g50**	**7**	**27**	**0.866**	**0.866**	**0.929**	**1240**	**1300**	**2540**
RI-set3_nofpkm	6	67	0.72	0.72	0.789	2834	3000	5834
RI-set3_fpkm	1	68	0.839	0.839	0.919	2834	3000	5834
RI-set3	6	68	0.901	0.9	0.964	2834	3000	5834
RI-set3_g9	6	60	0.907	0.907	0.962	2108	2100	4208
RI-set3_g20	5	61	0.867	0.867	0.939	1850	1900	3750

RI-set3_g30	6	44	0.876	0.875	0.919	1625	1600	3225
RI-set3_g40	6	30	0.874	0.873	0.944	1370	1400	2770
**RI-set3_g50**	**5**	**29**	**0.929**	**0.929**	**0.952**	**1188**	**1200**	**2388**
RI-set4_nofpkm	6	66	0.734	0.731	0.787	2825	3000	5825
RI-set4_fpkm	1	66	0.832	0.832	0.911	2825	3000	5825
RI-set4	6	66	0.88	0.879	0.951	2825	3000	5825
RI-set4_g10	6	45	0.886	0.886	0.952	1808	1800	3608
RI-set4_g20	6	40	0.884	0.884	0.94	1582	1500	3082
RI-set4_g30	6	35	0.891	0.891	0.958	1359	1400	2759
RI-set4_g40	5	24	0.899	0.899	0.958	1171	1200	2371
**RI-set4_g50**	**5**	**24**	**0.907**	**0.907**	**0.951**	**1047**	**1100**	**2147**
RI-set5_nofpkm	6	55	0.693	0.69	0.745	2346	2500	4846
RI-set5_fpkm	1	55	0.821	0.821	0.891	2346	2500	4846
RI-set5	6	56	0.868	0.866	0.945	2346	2500	4846
RI-set5_g10	7	50	0.899	0.899	0.952	1762	1800	3562
RI-set5_g20	7	48	0.888	0.887	0.947	1608	1600	3208
RI-set5_g30	6	36	0.861	0.86	0.95	1401	1400	2801
RI-set5_g40	6	25	0.863	0.861	0.912	1196	1200	2396
**RI-set5_g50**	**5**	**30**	**0.88**	**0.879**	**0.935**	**1063**	**1100**	**2163**
RI-set6_nofpkm	6	55	0.727	0.725	0.798	2298	2500	4798
RI-set6_fpkm	1	55	0.827	0.827	0.890	2298	2500	4798
RI-set6	6	55	0.896	0.895	0.957	2298	2500	4798
RI-set6_g10	6	47	0.874	0.873	0.926	1581	1600	3181
RI-set6_g20	5	43	0.875	0.875	0.944	1398	1400	2798
RI-set6_g30	6	25	0.872	0.872	0.947	1231	1200	2431
RI-set6_g40	6	22	0.854	0.854	0.936	1063	1000	2063
**RI-set6_g50**	**5**	**20**	**0.903**	**0.902**	**0.957**	**885**	**900**	**1785**
RI-set-stage-expressed_nofpkm	7	50	0.787	0.787	0.873	2443	2500	4943
RI-set-stage-expressed_fpkm	1	50	0.826	0.826	0.884	2443	2500	4943
RI-set-stage-expressed	7	50	0.903	0.903	0.976	2443	2500	4943
RI-set-stage-expressed_g10	7	27	0.933	0.933	0.979	1477	1500	2977
RI-set-stage-expressed_g20	7	25	0.922	0.922	0.976	1227	1200	2427
RI-set-stage-expressed_g30	7	20	0.916	0.916	0.967	1050	1100	2150
RI-set-stage-expressed_g40	7	17	0.931	0.931	0.971	851	900	1751
**RI-set-stage-expressed_g50**	**7**	**15**	**0.935**	**0.935**	**0.983**	**686**	**700**	**1386**
RI-set-all-expressed_nofpkm	6	28	0.670	0.670	0.725	968	1000	1968
RI-set-all-expressed_fpkm	1	28	0.721	0.721	0.791	968	1000	1968
RI-set-all-expressed	6	30	0.766	0.766	0.812	968	1000	1968
RI-set-all-expressed_g10	8	16	0.717	0.716	0.825	297	300	597
RI-set-all-expressed_g20	7	15	0.829	0.829	0.906	215	200	415
RI-set-all-expressed_g30	6	16	0.703	0.705	0.820	170	200	370

RI-set-all-expressed_g40	8	16	0.857	0.856	0.862	127	150	277
**RI-set-all-expressed_g50**	**8**	**12**	**0.944**	**0.944**	**0.955**	**84**	**100**	**184**

Table S3. The values of $\alpha(x(k))$ and $S(x(k))$ ($S_{True}(x(k))$ or $S_{False}(x(k))$) of some typical frequent motifs in the RI-set-all-expressed.

**motifs**	$\alpha(x(k))$	$S_{True}(x(k))$	$S_{False}(x(k))$
**cgga**	-0.567042475	0.130165289	0.076083236
**ggag**	-0.529287086	0.18285124	0.129440756
**tggag**	-0.522605671	0.115702479	0.075142036
**gagg**	-0.482489067	0.165289256	0.122390307
**cgg**	-0.439528941	0.28822314	0.209271682
**caag**	-0.423900818	0.128099174	0.089174481
**accg**	-0.415138539	0.120867769	0.081217058
**aagag**	-0.402469414	0.142561983	0.097628174
**tcgg**	-0.360944905	0.143595041	0.104233692
**cggt**	-0.374540209	0.13946281	0.104011226
**cattt**	0.305908101	0.246900826	0.35053734
**tattt**	0.34198048	0.299586777	0.438856185
**atttt**	0.262753068	0.405991736	0.542388254
**tata**	0.250785518	0.459710744	0.579591348
**aattt**	0.27681664	0.303719008	0.419997946
**tttta**	0.231170772	0.359504132	0.47075433
**ttttt**	0.225772566	0.439049587	0.557224998
**atat**	0.247623358	0.490702479	0.618197686
**tagt**	0.22940222	0.354338843	0.444588952
**tatt**	0.220892226	0.558884298	0.709990417

Table S4. The values of $\alpha(x(k))$ and $S(x(k))$ ($S_{True}(x(k))$ or $S_{False}(x(k))$) of some typical frequent motifs in the RI-set-stage-expressed_g50 (RIg50 for short).

**motifs**	$\alpha(x(k))$	$S_{True}(x(k))$	$S_{False}(x(k))$
**cgg**	-0.816768402	0.521865889	0.209271682
**ccg**	-0.784297556	0.549562682	0.224074201
**agga**	-0.783093964	0.486880466	0.196060648
**gcg**	-0.775286709	0.470845481	0.194007119
**gaag**	-0.755469722	0.54664723	0.267968376
**agag**	-0.701591933	0.478134111	0.239698131
**cgc**	-0.699649506	0.451895044	0.205660894
**caga**	-0.673699882	0.457725948	0.232938599
**ggaa**	-0.664631609	0.513119534	0.262800329
**ggg**	-0.650464117	0.574344023	0.282736669
**agg**	-0.649087525	0.728862974	0.411441577

**caag**	-0.645785664	0.508746356	0.263844206
**gaga**	-0.639515896	0.495626822	0.277414607
**ggc**	-0.616765671	0.593294461	0.329334657
**cga**	-0.616664842	0.655976676	0.366948456
**gcc**	-0.603552829	0.574344023	0.313163119
**gga**	-0.588726833	0.790087464	0.519440071
**acg**	-0.563773743	0.577259475	0.335375453
**aagc**	-0.561489245	0.459183673	0.276764323
**gag**	-0.554618971	0.7696793	0.558285988
**ttctt**	0.463526261	0.422740525	0.553443083
**gtttt**	0.474747218	0.39212828	0.527483058
**ttgtt**	0.480745417	0.403790087	0.54498939
**tata**	0.485435797	0.441690962	0.579591348
**tcttt**	0.493161333	0.376093294	0.53099117
**tttct**	0.495520556	0.415451895	0.573550551
**ttttg**	0.516373908	0.416909621	0.576699295
**tgttt**	0.531488491	0.408163265	0.582586077
**tttgt**	0.53605445	0.393586006	0.550431241
**tttt**	0.537270663	0.638483965	0.874563625
**ttttc**	0.538606957	0.371720117	0.517283866
**atttt**	0.56041678	0.373177843	0.542388254
**ctttt**	0.563811027	0.33819242	0.501967965
**ttttt**	0.629014696	0.336734694	0.557224998

Table S5(Table S5.xlsx). The 79 KEGG enrichment pathways involving the genes which contain co-occurring RIs in multiple samples.
