# Supplementary material for: 50/50 Expressional Odds of Retention Signifies the Distinction between Retained Introns and Constitutively Spliced Introns in Arabidopsis thaliana
Source: Front Plant Sci. 2017 Oct 9;8:1728. doi: 10.3389/fpls.2017.01728 (PMC5640774; doi:10.3389/fpls.2017.01728)

1) AT5G37370 (ATSRL1)

Gene name		AT5G37370
Chromosome name		Chr5
start		14815844
end		14815937
strand		-
Sample1	FPKM of RI-containing isoforms	42.02661
	FPKM of isoforms without RIs	3.433038
Sample2	FPKM of RI-containing isoforms	70.73547
	FPKM of isoforms without RIs	0.808133
Sample3	FPKM of RI-containing isoforms	31.42164
	FPKM of isoforms without RIs	1.969896
Sample4	FPKM of RI-containing isoforms	55.83697
	FPKM of isoforms without RIs	4.087963
Sample5	FPKM of RI-containing isoforms	46.68571
	FPKM of isoforms without RIs	6.947562
Sample6	FPKM of RI-containing isoforms	64.00962
	FPKM of isoforms without RIs	6.709976

TAIR10 AT5G37370.1/AT5G37370.2/AT5G37370.3 ⟷ AT5G37370.4

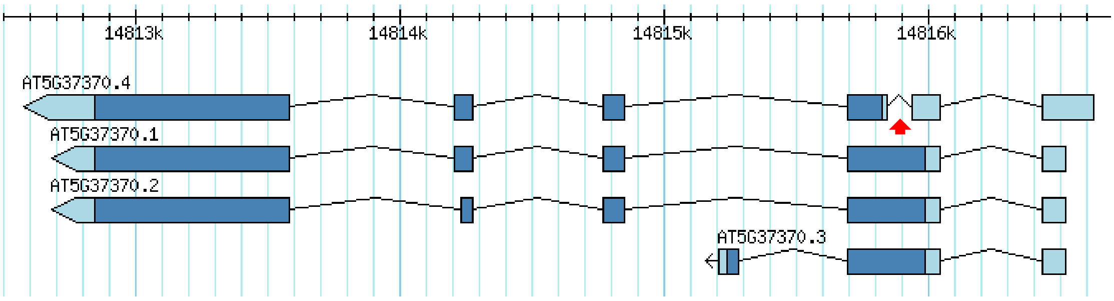

Sample12 TCONS_00054773/TCONS_00054775/TCONS_00054776/TCONS_00054777

⟷ TCONS_00054771/TCONS_00054772/TCONS_00054774

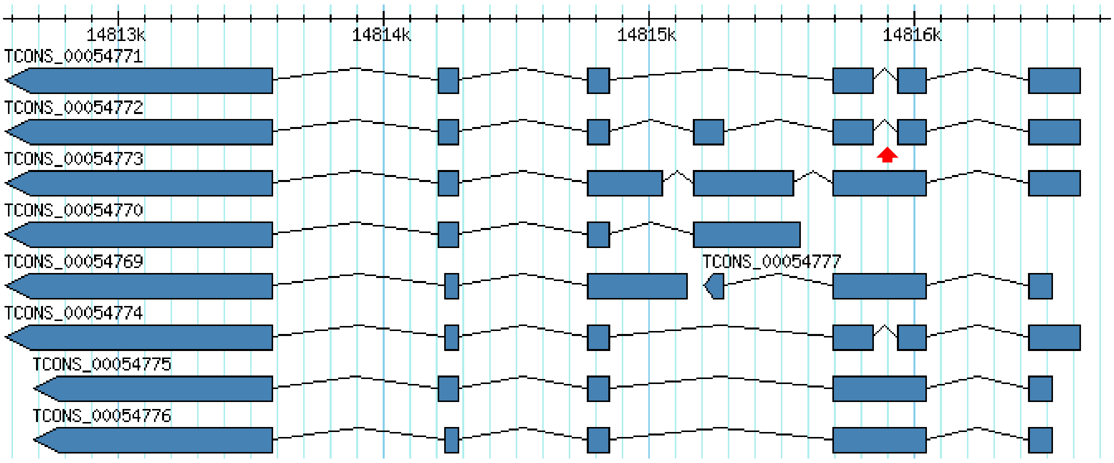

Sample34 TCONS_00043120/TCONS_00043121/TCONS_00043122 ⟷

TCONS_00043118/TCONS_00043119/TCONS_00043123

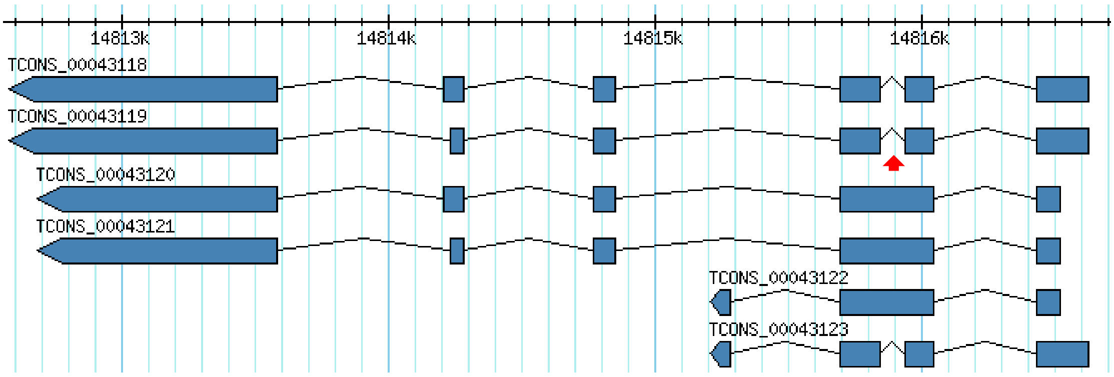

Sample56 TCONS_00042609/TCONS_00042610/TCONS_00042611 ⟷ TCONS_00042607/TCONS_00042608/TCONS_00042612

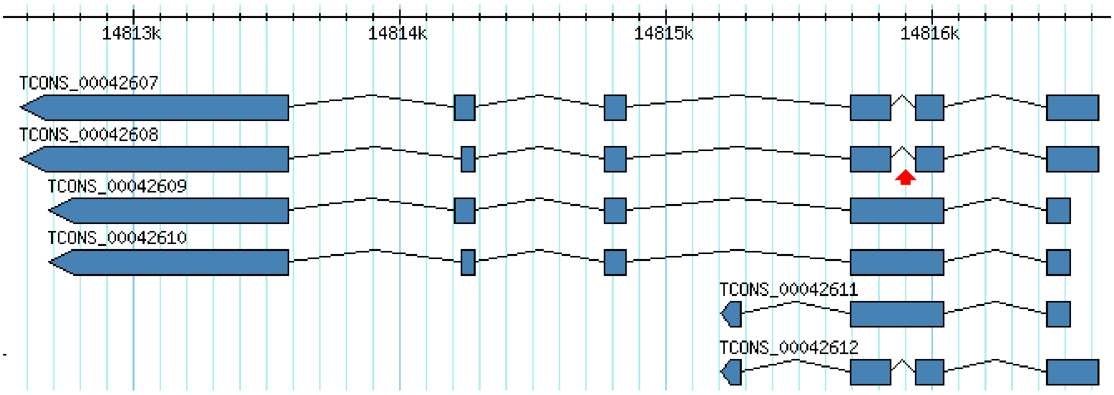

2) AT1G55310 (ATSCL33)

Gene name		AT1G55310
Chromosome name		Chr1
start		20631120 or 20631723
end		20631686 or 20631884
strand		+
Sample1	FPKM of RI-containing isoforms	5.65144
	FPKM of isoforms without RIs	3.925671467
Sample2	FPKM of RI-containing isoforms	23.9866
	FPKM of isoforms without RIs	16.62581041

Sample12 TCONS_00005042 ⟷ TCONS_00005043/TCONS_00005044

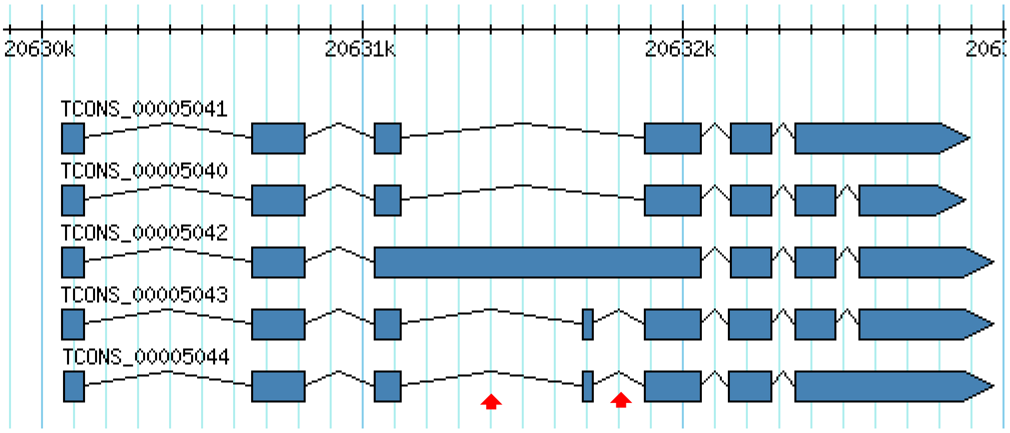

The intron(20631723-20631884, Chr1) does not retain in TAIR10, Sample34 and Sample56

TAIR10

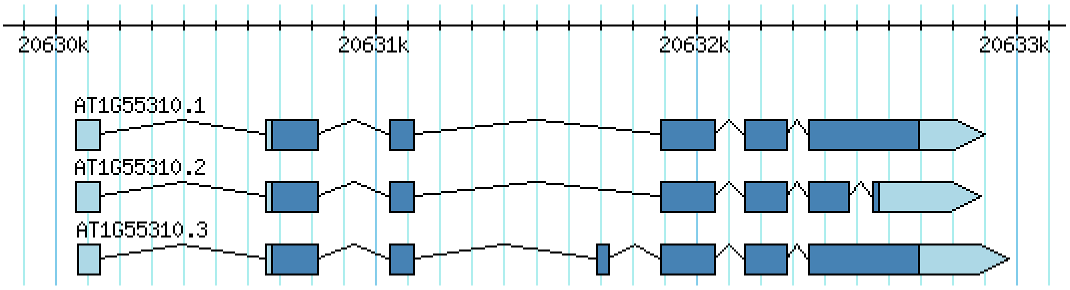

Sample34

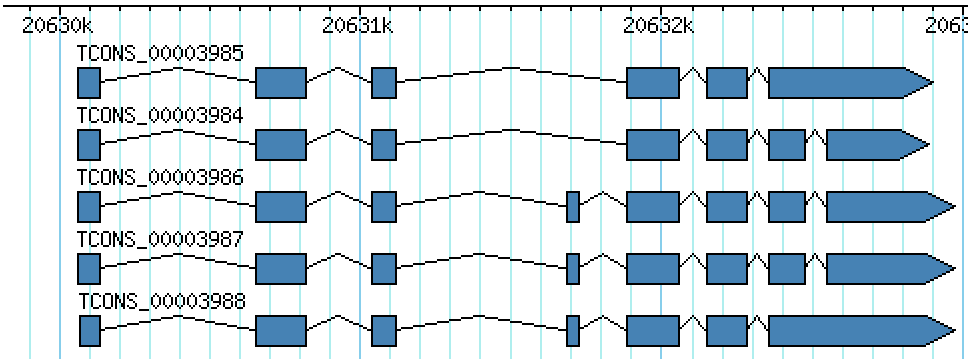

Sample56

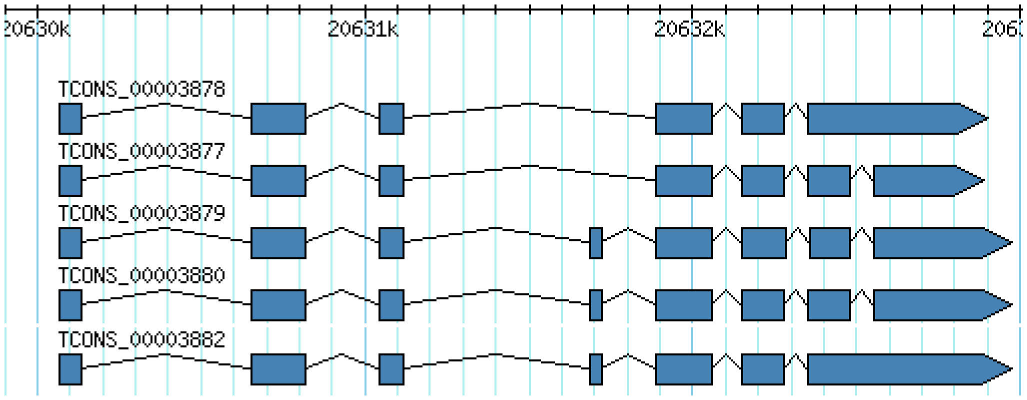

3) AT1G28060 (RDM16)

Gene name		AT1G28060
Chromosome name		Chr1
start		9779826
end		9779904
strand		+
Sample1	FPKM of RI-containing isoforms	10.4971
	FPKM of isoforms without RIs	2.73047
Sample2	FPKM of RI-containing isoforms	13.7793
	FPKM of isoforms without RIs	9.50708

Sample12 TCONS_00002760 ⟷ TCONS_00002758

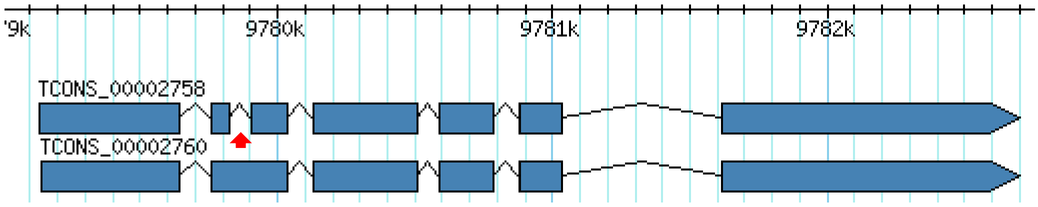

The intron(9779826-9779904, Chr1) does not retain in TAIR10, Sample34 and Sample56

TAIR10

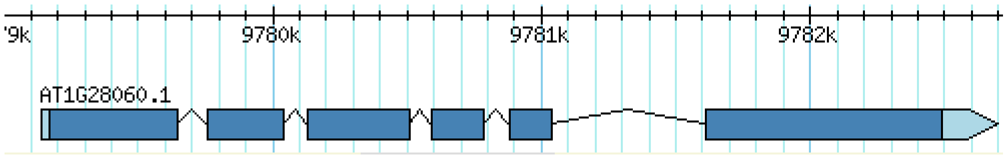

Sample34

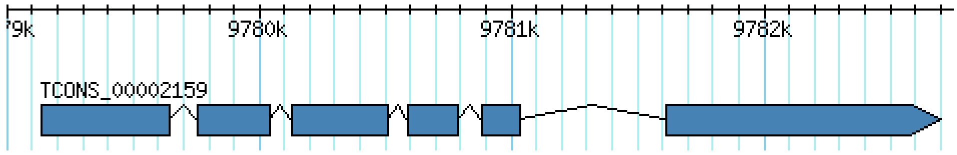

Sample56

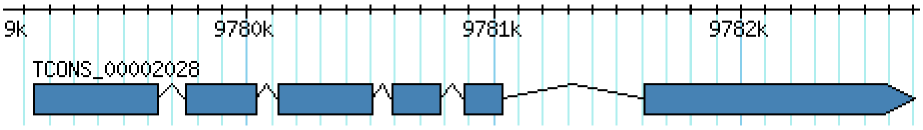

4) AT4G14300 (DL3190W)

Gene name		AT4G14300
Chromosome name		Chr4
start		8232878
end		8233099
strand		+
Sample1	FPKM of RI-containing isoforms	40.6679
	FPKM of isoforms without RIs	1.88458
Sample2	FPKM of RI-containing isoforms	25.9766
	FPKM of isoforms without RIs	12.6302

Sample12 TCONS_00037512 ⟷ TCONS_00037511

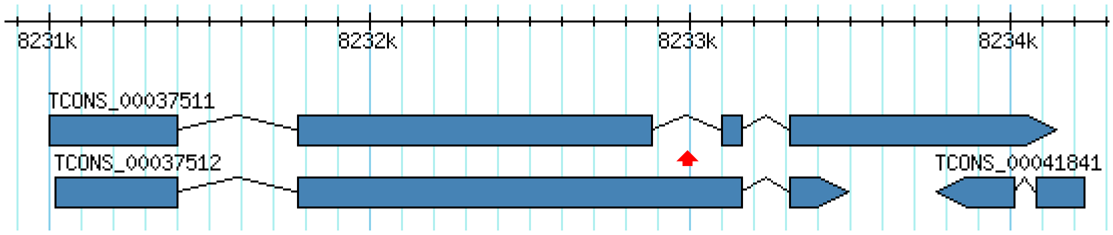

The intron(8232878-8233099, Chr4) does not retain in TAIR10, Sample34 and Sample56

TAIR10

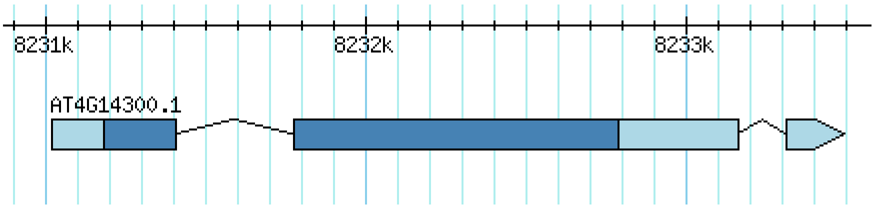

Sample34

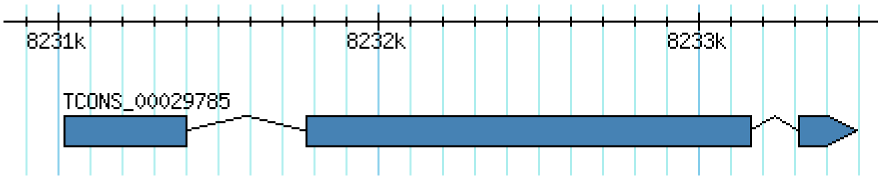

Sample56

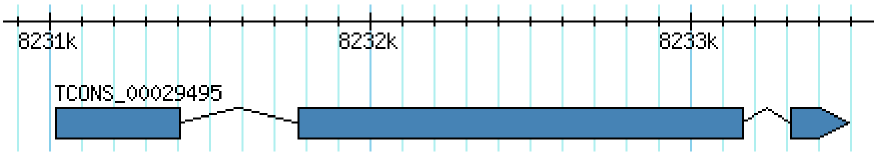

5) AT2G13540 (ABH1)

Gene name		AT2G13540
Chromosome name		Chr2
start		5641494
end		5641543
strand		+
Sample1	FPKM of RI-containing isoforms	13.75963
	FPKM of isoforms without RIs	0.26652
Sample2	FPKM of RI-containing isoforms	31.448523
	FPKM of isoforms without RIs	0.000844279

Sample12 TCONS_00016108/TCONS_00016110 ↔ TCONS_00016109

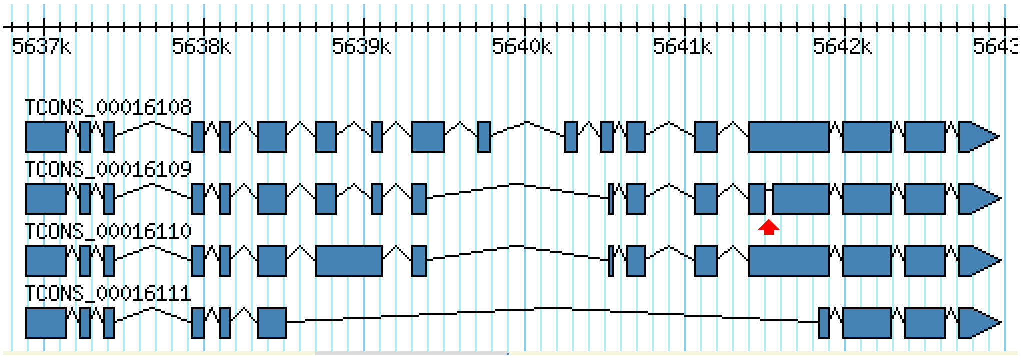

The intron(5641494-5641543, Chr2) does not retain in TAIR10, Sample34 and Sample56

TAIR10

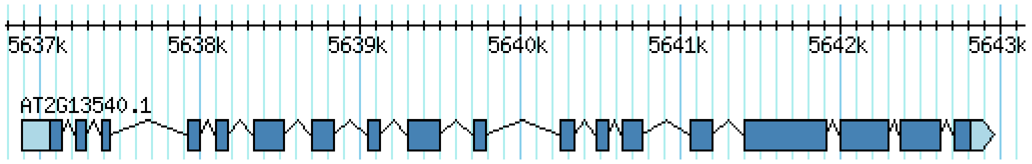

Sample34

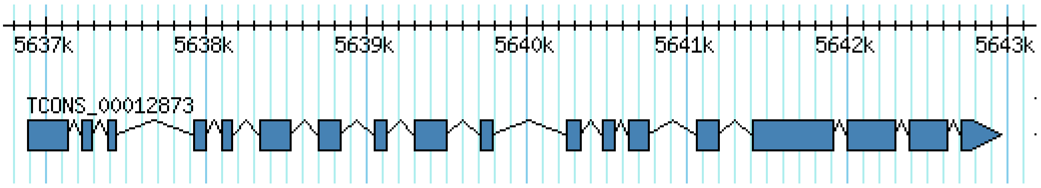

Sample56

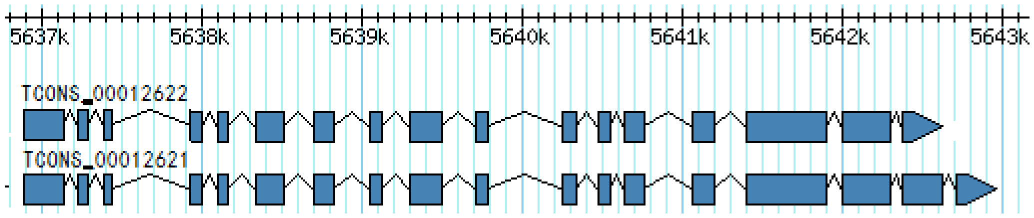

Supplement: Supplementary file 6 [file DataSheet4.PDF]
